# Recent Progress on the Synergistic Antitumor Effect of a Borneol-Modified Nanocarrier Drug Delivery System

**DOI:** 10.3389/fmed.2021.750170

**Published:** 2021-11-25

**Authors:** Jinxiu Li, Qian Xie, Rong Ma, Yong Li, Jianmei Yuan, Mihong Ren, Hongyan Li, Jiajun Wang, Danni Lu, Zhuo Xu, Jian Wang

**Affiliations:** ^1^State Key Laboratory of Southwestern Chinese Medicine Resources, Chengdu, China; ^2^College of Pharmacy, Chengdu University of Traditional Chinese Medicine, Chengdu, China

**Keywords:** borneol, nanocarrier, drug delivery, targeting, drug resistance, tumor therapy

## Abstract

Borneol, a traditional Chinese medicine, can enhance therapeutic efficacy by guiding the active ingredients to the target site. Reportedly, borneol improves the penetration capacity of the nasal, cornea, transdermal, intestinal, and blood-brain barriers. Although nanotechnology dramatically changed the face of oncology by targeting tumor sites, the efficiency of nanoparticles delivered to tumor sites is very low, with only 0.7% of the total particles delivered. Thus, based on the penetration ability and the inhibition drug efflux of borneol, it was expected to increase the targeting and detention efficacy of drugs into tumor sites in nanocarriers with borneol modification. Borneol modified nanocarriers used to improve drug-targeting has become a research focus in recent years, but few studies in this area, especially in the antitumor application. Hence, this review summarizes the recent development of nanocarriers with borneol modification. We focus on the updated works of improving therapeutic efficacy, reducing toxicity, inhibiting tumor metastasis, reversing multidrug resistance, and enhancing brain targeting to expand their application and provide a reference for further exploration of targeting drug delivery systems for solid tumor treatment.

## Introduction

Malignant tumors are one of the most devastating diseases, seriously affecting human life worldwide. Presently, the main treatment of cancer involves surgical resection, radiotherapy, chemotherapy, or immunotherapy, much of which is often insufficient and can cause serious, burdensome, and undesirable side effects (1, 2). Moreover, some chemotherapeutics, such as paclitaxel (PTX) or doxorubicin, have a low solubility in water, resulting in poor preferential absorption in the gastrointestinal tract (GIT), lack of distribution selectivity, first-pass metabolism, and overexpression of drug efflux transporter pumps [e.g., P-glycoprotein (P-gp)], which reduce their bioavailability and limit their therapeutic effects. Hence, the therapeutic effect of a single drug is limited. Moreover, it has been reported that cancer becomes more heterogeneous with its progression, which further leads to the formation of a group of cancer cells, which, together with changes in tumor cell proliferation, invasion, and migration decreases the sensitivity, tumor recurrence, and adverse reactions (3, 4). Multidrug resistance (MDR) is a phenomenon that occurs when cancer cells become resistant to multiple chemotherapeutic drugs with distinct structures and functions and remain a major hurdle to the successful treatment of various types of cancer (5). For a long time, the combination of anticancer drugs improved drug resistance and therapeutic effect (6). However, the different pharmacokinetics and lack of efficient drug delivery systems have greatly weakened their therapeutic effects.

**Figure 1:**
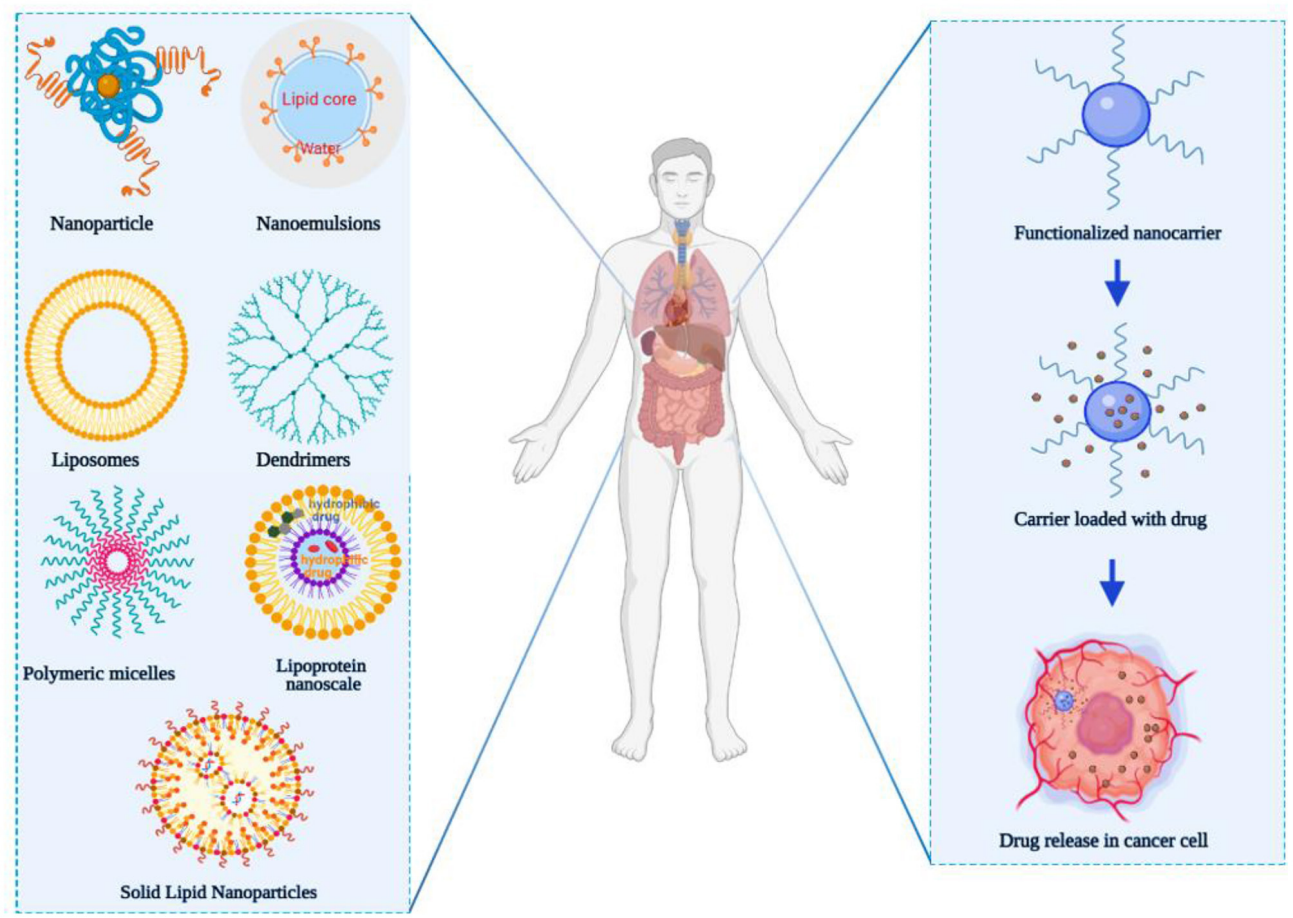
Major classes of nanocarrier utilized for overcoming multidrug resistance (nanoparticle, nanoemulsions, liposomes, dendrimers, polymeric micelles, lipoprotein nanoscale, and solid lipid nanoparticles). The figure was created by BioRender, https://app.biorender.com/.

Nanocarriers for drug delivery offer a novel idea for cancer treatment and have inspired many researchers. Nanocarriers can increase the concentration of a drug at the tumor site *via* the enhanced permeability and retention (EPR) effect, which also reduces systemic toxicity (7–10). This phenomenon was first discovered by Matsumura and Maeda in 1986 when they observed that macromolecules >50 kDa preferentially distributed to the tumor tissues compared to normal tissues (11). In recent years, various nanocarriers, with different diameter, structures, and surface properties, including liposomes, polymer micelles, nanoparticles (NPs), dendrimers, nanoemulsions, and lipoprotein nanocomposites (Figure 1) have been designed for the specific delivery of anticancer drugs toward the tumor site (12–14). At present, some nanomedicines have been used in the clinic such as Doxil® (polyethylene glycol-modified liposome adriamycin) (15), micellized paclitaxel nanodrug Genexol®-PM injection (16), and liposome vincristine Marqibo® (17). When nanocarriers interact with biological systems barriers, such as the skin epithelial barrier, the GIT, and the air-blood barrier, they serve as part of the primary defense system. These barriers prevent nanocarriers access into the deeper levels of organs affect the percentage of NPs that reach the diseased target tissue and cells (18). Biological barriers generally use common mechanisms to regulate the access of nanocarriers, whereas new mechanisms can be developed to block nanocarriers, especially in the tumor microenvironment, thereby further increasing the complexity of their behavior (19, 20). Additionally, NPs in the tumor matrix will also be affected by the cell-to-cell transport, enhanced permeability, retention effects, and may also compete with organs. Thus, the NPs delivered into tumor sites only account for 0.7% of the administered dose, which greatly hinders the clinical transformation of nanomedicine (21). Hence, how to solve the problem of NP delivery and its absorption and accumulation in tumor sites is extremely important. Borneol can promote the transdermal absorption of insoluble drugs and increase the blood concentration and bioavailability of drugs. Therefore, the introduction of borneol in nanocarriers is expected to increase the transmembrane absorption and the delivery volume to tumor tissues.

Borneol (*Bing-Pian* or *Long-Nao*) is a representative traditional Chinese medicine (TCM) resuscitation drug that has been used for more than 1,500 years. Borneol (C_10_H_18_O, molecular weight 154.25 Da) is a highly lipid-soluble bicyclic monoterpene with a fragrant odor and pungent and bitter taste. Borneol is a promising candidate for promoting the absorption of certain drugs and is generally used as a brain-refreshing drug in TCM. The borneol can be divided into the following three categories due to the differences in sources: natural borneol (NB, d-Borneol), levorotatory borneol (l-Borneol), and synthetic borneol (SB). Synthetic borneol comprises d-borneol and isoborneol, whereas natural borneol contains only d-borneol (22). Because some studies do not illustrate which type of borneol was utilized, we will simply use “borneol” in this manuscript. Borneol is widely regarded as a messenger drug that is considered capable of introducing main effective drugs in the prescription to the target site to increase therapeutic efficacy (23). This is consistent with the theory that “borneol is not potent enough when used alone; when it is used as an assistant or a messenger, borneol is effective” as described in the “Extension of the Materia Medica (Ben Cao Yan Yi)” (24). Moreover, borneol can promote the transdermal absorption of other drugs and increase their blood concentration and bioavailability (25). Therefore, it is a promising penetration enhancer based on its strong permeabilization effect. In addition, borneol can also prevent the efflux of drugs by inhibiting the function of P-gp and consequently increasing the accumulation of drugs and improving the therapeutic effect (26). Regarding the use of penetration enhancers, for instance, Azone was proved to promote transdermal penetration of both lipophilic and hydrophilic drugs. Furthermore, Azone, dimethyl sulfoxide (DMSO), decamethonium, glycocholate, and cholate are also used as ocular penetration enhancers in the laboratory. These penetration enhancers will easily generate irritability and toxicity, and the applicable concentrations are generally lower than those used on the skin owing to the corneal epithelium and the keratoderma being different in cellular type, pharmacokinetics, and metabolic processes and the cornea being sensitive to a chemical stimulus of foreign materials. For example, 0.1% Azone or a slightly higher concentration can cause ocular hypersensitivity, discomfort, or toxicity (27). Furrer et al. compared the ocular tolerance of nine potential absorption promoters, results indicated that DMSO, decamethonium, Tween 20, Brij 35, EDTA, glycocholate, and cholate caused about 16% of the corneal surface damaged, while sodium fusidate and saponin caused over 30% injured of the cornea (28). Surfactants (for example, ethanol and propylene glycol) are added to formulations to solubilize lipophilic active ingredients, but anionic and cationic surfactants have the potential to damage human skin (29). In the “National Collection of Chinese Medicine Products” of China, there are 76 ophthalmic preparations, 73% of which contain borneol. Borneol could open the tight junctions and improve the permeability of the blood-optic nerve barrier reversibly by regulating the expression of claudin-5 and occluding (30). It has been noted that the opening of blood-brain barrier (BBB) by borneol was rapid, reversible, and causes transient physiological opening, which ensures the structural integrity of BBB (31). Borneol can be considered safe for oral consumption and U.S. Food and Drug Administration has listed borneol as a food ingredient. Natural and synthetic borneol at a concentration of 0.1% was safe for ocular administration and repeated administration for a long time without irritation, inflammation, sensitization, or corneal tissue damage (32, 33). These reports show that borneol not only has a great penetration enhancement effect, but is also with high safety and easy availability due to its low cost (34–36). Thus, the application of borneol in nanocarriers has great potential to improve the targeting and enhance the accumulation of chemotherapeutic drugs in tumors.

Here, this review first describes the recent research progress of borneol in combination with nanocarriers such as NPs, nanoemulsions, liposomes, dendrimers, polymer micelles, and lipoprotein nanocomposites for improving therapeutic efficacy, reducing the toxicity, inhibiting tumor metastasis, reversing MDR, and enhancing brain targeting in solid tumor treatment. Therefore, our review provides a theoretical reference for the further application of borneol in nanocarrier drug delivery systems.

## Penetration Promoting Effect and Mechanism of Borneol

Borneol is a cyclic terpene alcohol, and several studies have shown that it can enhance the diffusion of certain drugs across various physiologic barriers, such as the BBB (37), the cornea (38), the skin (39), and the mucous membrane (40, 41), indicating that borneol can be used as a transdermal penetration enhancer.

### Blood-Brain Barrier (BBB)

It is reported that borneol can easily and rapidly cross the BBB due to its low molecular and high lipid solubility. The opening is a rapid, reversible, and transient physiological opening which causes no structural damage (31). Therefore, it is used as an adjuvant owing to its permeation-enhancing effects on various drugs across the BBB to treat central nervous system diseases. BBB is a special barrier between blood and brain tissue, which includes a structural barrier and a functional barrier. The structural barrier comprises tight junctions between vascular endothelial cells, endothelial cell-cell junctions, basement membrane, and astrocytes (42). The functional barrier is mainly comprising ATP-binding cassette (ABC) transporters including P-gp, multidrug resistance proteins 1a and 1b (MDR 1a and MDR 1b), multidrug resistance-associated protein 1 (MRP 1), MRP 4, and MRP 5, and the breast cancer resistance proteins (BCRP), among which P-gp is the most in-depth study. P-gp is a drug efflux pump, which can prevent the entry of drugs from blood into the brain (22, 43). The permeability-enhancing effects of borneol are closely associated with the inhibition of efflux protein function (44). In addition, borneol opens the tight junctions in BBB by modulating the expression of proteins of proteins like claudin and occluding (45). Borneol may also enhance pinocytosis in brain capillary endothelial cells, thereby promoting the transcellular passage of drug moieties. Taken together, alteration in cell membrane lipid structures, modulation of multiple ATP-binding cassette transporters, and tight junction proteins are the major contributing factors to BBB opening functions of borneol.

It has been shown that synthetic borneol maintained the cisplatin in the mouse brain at high concentrations for a long duration. The mechanism was that borneol increased the expression of ZO-1 and intercellular cell adhesion molecule-1 (ICAM-1), and inhibited the expression of P-gp (44). The intraperitoneal injection of cisplatin after oral administration of L-borneol promoted the entry of cisplatin into brain tissue. The mechanism was that borneol promoted the opening of the BBB by the loss of tight junction proteins including occluding and claudin 5 at 1 h after the intravenous injection of L-borneol (45). Moreover, a borneol/menthol eutectic mixture promoted the transport of cobalt toxin into the brain by coadministration with borneol through the nasal-brain pathway by opening the tight junctions between the cells (46). Oral administration of borneol at different intervals has also been shown to increase the quantity and velocity of geniposide permeating in the brain, especially after 15 min of intragastric borneol at which point the BBB is most open (47). Borneol enhanced the antidepressant effects of asiaticoside by promoting its penetration of the BBB, thus enhancing the anti-depressant effects with enhanced 5-HT and BDNF, and reduced TNF-α levels (48). Furthermore, the brain bioavailability of geniposide was found to be enhanced effectively. The possible mechanism may be that borneol could bind to the site on the cellular membrane or adsorb to the membrane surface to improve the membrane fluidity of the epithelium, increasing the orderly arrangement of cellular membrane phospholipid molecule chains, and reducing the collision with phospholipid molecules when a drug crosses the membrane. Thereby facilitating drug permeation. The levels of the tight junction (TJ) proteins localized in cell-cell junctions were decreased when cells were exposed to borneol, suggesting an impairment of the intercellular junctions, and increased paracellular permeability (49) (Table 1).

**Table 1:** The penetration promoting effect of borneol in brain, corneal, nasal mucosa, skin, and gastrointestinal mucosa.

**Penetration target site**	**Drug**	**Absorption enhancer**	**Administration**	**Index**	**Mechanism**	**References**
Brain	/	10% borneol corn oil	Intragastrical	Occludin, ZO-1, NOS, P-gp, ICAM-1	Increased ICAM-1; opening of the BBB	(44)
	Cisplatin	L-borneol	Intragastrical administration	Evans blue leakage, cisplatin concentrations in peritumoral tissue and tumor loci, the median survival period, Occluding, Claudin-5	Loosen the intercellular tight junction in the BBB	(45)
	Cobrotoxin	Borneol/menthol eutectic mixture (+BMEM)	Intranasal administration	AUC; prolonged time values to peak concentrations	Loosen the tight junctions between the cells	(46)
	Geniposide	Borneol	Borneol: intragastric administration geniposide: iv	C_max;_ AUC; mean residence time (MRT); T_max_	BBB opening	(47)
	Asiaticoside	Borneol	Intragastric administration	Increased rat brain AS levels, BDNF, 5-HT; Decreased TNF-α	Opening the BBB	(48)
	Geniposide	Borneol and muscone	/	Enhanced permeability- effect of MDCK-MDR1 cells; increased the fluxes of geniposide in both directions in a concentration-dependent manner	TJ protein decreased	(49)
Corneal	Puerarin eye drops and timolol maleate eye drops	Borneol	/	Q: the accumulated release amount within time t, the homeostasis flow rate J, permeability parameter Kp	/	(32)
	Indomethacin, dexamethasone	Synthetic borneol and natural borneol	/	The apparent permeability coefficient (Papp), level of corneal hydration,	Loosen the tight junctions between epithelial cells	(33)
	Danshensu	Borneol	Intragastric administration	Danshensu concentrations in plasma, aqueous humor, and vitreous humor, peak times (Tmax),	/	(30)
	/	Synthetic borneol	Borneol gastric lavage	Claudin-5 and occluding in endothelial cells distribution and the mRNA, protein level	Translocation of claudin-5 and occluding	(34)
	Fluconazole	Menthol and borneol	/	Fluconazole concentration assay; the apparent corneal permeability coefficient (Papp); the transport enhancement ratios (ER); corneal hydration levels	Improved the membrane fluidity; reduced the collision with phospholipid molecules	(35)
	Rhodamine B, sodium-fluorescein, fluorescein isothiocyanate (FITC)	Borneol	/	Increased the apparent permeability coefficient (Papp) and Draize score, maintained the corneal hydration values <83%; enhanced the drugs into the eye	Loosen the corneal epithelium junctions reversibly	(50)
	Geniposide (Ge)	Natural borneol	Ophthalmic administered	Concentrations of Ge; apparent permeability coefficient (Papp); AUC_0−6*h*_; C_max_	NB enhanced epithelial junction permeability and promoted paracellular drug absorption.	(36)
Skin	5-fluorouracil, antipyrine, aspirin, salicylic acid and ibuprofen	Borneol	Transdermal permeation	Irritant profile analyzed by transepidermal water loss (TEWL); molecular organization of SC lipids detected by ATR-FTIR	Interact or associate with the SC lipids alkyl chains	(51)
	Transdermal drugs	Borneol and menthol	Transdermal permeation	Decreased the expression of MDR1and P-gp; reduced Rh123 efflux; increased Rh123 accumulation	Inhibit MDR1and P-gp	(52)
	5-fluorouracil	Borneol and menthol	*In vitro* permeation	Disrupted SC morphology; induced a slight curvature in the SC bilayer; increased diffusion coefficient of 5-FU	Menthol: disruption of the stratum corneum (SC) bilayer borneol: the disruption of the SC bilayer, increased the diffusion, induced the formation of transient pores	(53)
Gastrointestinal mucosa	Notoginsenoside R1 (NGR1), Ginsenosides Rg1 (GRg1) and Re (GRe)	Borneol	Intragastric administration	Increased bioavailability, the quantities of NGR1, GRg1, and GRe in rabbit tissues and *P*app	Loose intercellular tight junction	(54)
	Curcumin	Borneol	Intragastric administration	Increased AUC _0−t_, C_max_ and *P*app	No description	(55)
Nasal mucosal	Geniposide (Ge)	Borneol and muscone	/	Immunostaining of TJ proteins; decreased transepithelial electrical resistance (TEER); increased apparent permeability coefficient (Papp) and fluorescence recovery rate (R); efflux ratio (ER);	Reduce drag of phospholipids of the lipid bilayer in the nasal epithelium; loose intercellular tight junction; increase the number and volume of pinocytosis esicles in BBB cells	(56)

In recent time, some studies have been conducted to explore the potential of combining borneol with various nanocarriers to improve pharmacokinetics along with the dynamics of the drugs toward the brain. Borneol also increased the uptake of NPs when borneol and aprotinin-conjugated poly (ethylene glycol)-poly (L-lactic-co-glycolic acid) NPs (Apr-NP) were cocultured with brain capillary endothelial cells (BCECs), suggesting that borneol enhanced NPs delivery into the brain by loosening the intracellular tight junction in BBB and accelerating the transportation of substance through the intercellular passage (57). Moreover, lactoferrin (Lf) co-modified NPs with borneol modification (Lf-BNPs) increased cellular uptake of 16HBE cells *in vitro*. Borneol and Lf co-modified NPs not only enhance the penetration and transport of dopamine through the nasal mucosa, but also enhance the drug delivery by opening the tight junctions of the BBB (58). There are two main methods for borneol to modify the carrier: the physical mixing method (physical modification) and the chemical synthesis method (chemical modification). At present, most of the modification are the physical modification, which mixed borneol and other lipophilic ingredients physically. The temperature needs to be controlled during the process owing to the sublimation of borneol. The chemical modification is borneol covalently combined with lipid materials based on a hydroxyl group structure of borneol through a certain chemical reaction. Song et al. compared the enhancing efficiency of solid lipid NPs between chemical (BO-SLN/CM) or physical modification with borneol (BO-SLN/PM). The results demonstrated that BO-SLN/CM exhibited lower cytotoxicity, higher cell uptake, and better brain targeting when compared with BO-SLN/PM. In addition, BO-SLN/CM had a remarkable targeting ability to the brain, while BO-SLN/PM was concentrated at the lung (59). A study explored whether the borneol modified SLNs loaded with Pueraria flavone chemically (PTF-Bo-SA-SLNs) and physically (PTF-Bo-SLNs) improve brain delivery following intranasal administration. The results showed that both PTF-Bo-SA-SLNs and PTF-Bo-SLNs promoted the uptake of PTF by Caco-2 cells in a time and concentration-dependent manner, among them, the PTF-Bo-SA-SLNs showed better uptake especially at high concentrations, which may be associated with the directly conjugated borneol on the nanoparticle surface. In addition, PTF-Bo-SA-SLNs increased the AUC and C_max_ of PTF in the plasma to 4.44- and 4.41-fold compared with PTF-Bo-SLNs, which suggested borneol modification could improve the absorption of PTF into plasma. There are two main direct pathways of entry into the brain after intranasal administration, namely, the olfactory and the trigeminal pathway. PTF-Bo-SA-SLNs and PTF-Bo-SLNs can be transported directly from the nasal cavity into the brain through the olfactory region of the nasal epithelium and the trigeminal neural region avoiding the BBB (60). Furthermore, borneol co-modified liposomes loaded with palmitate azidothymidine have been shown to enhance the transportation of drugs to the brain and increase the accumulation of azidothymidine by 3.73-fold, indicating that the addition of 10% borneol enhances the transportation of drugs in the brain by loosening the tight junctions of BBB (61). Ganciclovir (GCV)-loaded solid lipid NPs with borneol (GCVb-SLN) enhanced the transport of GCV to the brain and extended the residence time is probably because of a combined function of SLNs and borneol. SLNs can inhibit the drug efflux, open the tight junctions of the brain capillary endothelial cells, and through the brain capillary endothelial cells endocytosis or transcytosis. Borneol markedly loosens the intercellular tight junctions in the BBB and accelerates the transport of substances through the intercellular passages, and significantly inhibited the activity of P-gp (62). Moreover, borneol and ginkgolide encapsulated liposomes (GGB-LP) enhanced the BBB permeability of ginkgolide and increased the uptake of bEND.3 cells, resulting in improved targeting and distribution in the brain tissue by loosening intercellular tight junctions, opening the BBB, and enhancing the distribution of drugs in the brain (63) (Table 2).

**Table 2:** Nanomedicine co-modification and co-administration with borneol can enhance drug delivery and absorption to the brain, skin, and gastrointestinal mucosa.

**Disease**	**Drug**	**Nanomedicines drug delivery system**	**Animal model**	**Cell**	**Administration**	**Action results**	**Mechanism**	**References**
**BBB**								
AD	Huperzine A (Hup A)	Aprotinin-conjugated poly (ethyleneglycol)-poly (Llactic-co-glycolic acid) nanoparticles (Apr-NP)	Balb/c nude mice; Sprague–Dawley (SD) rats	Mouse brain capillary endothelial cells (BCECs)	Injected by tail vein	Borneol could enhanced the cell uptake of nanoparticles Greatly and the distribution of the nanoparticles into the brain, improved the brain targeting efficiency of nanoparticles and memory impairment of AD rats.	Loose the intracellular tight junction	(57)
Parkinson's disease (PD)	Dopamine	Borneol and lactoferrin co-modified nanoparticles (Lf-BNPs) encapsulated dopamine	Male SD rats	SH-SY5Y, 16 HBE cells	Intranasal administration	Lf and borneol co-modified NPs significantly increased the cellular uptake and promoted dopamine delivery to the brain *via* the intranasal route, restored dopamine level in the lesioned striatum of 6-OHDA-induced PD rats	No description	(58)
/	/	Borneol-modified chemically solid lipid nanoparticle (BO-SLN/CM) and physically solid lipid nanoparticle (BO-SLN/PM)	Kunming mice	HBMECs cells	Injected *via* tail vein	Borneol significantly reduced the toxicity of SLN to HBMECs at high concentrations, improved the ability of cell migration, excellent enhancement of the BBB permeation, entered the brain rapidly	Improved in the fluidity of phospholipid molecules in the membrane, the tight junction disappeared, cells shrank, and the gap between cells increased	(59)
Cerebrovascular disease	Pueraria flavones (PTF)	PTF-loaded SLNs were modified with Bo by physical and chemical methods to synthesize PTF-Bo-SA-SLNs and PTF-Bo-SLNs	Male SD rats	Caco-2 cells	Intranasal administration	Modified with borneol increased the uptake of PTF-Bo-SA-SLNs and PTF-Bo-SLNs by Caco-2 cells, showed the better targeting effect, increased the (AUC), C_max_, and Bo modified by chemistry method is better than by physical method	No description	(60)
HIV encephalopathy	Azidothymidine palmitate	Azidothymidine palmitate liposomes with 10% borneol (10%B-AZTPL)	KM mice	/	Injected by tail vein	10%B-AZTPL significantly increased AZTP permeability into brain tissues, shortened the time to reach the peak and increased the peak concentration	Borneol loosen the tight junctions of BBB	(61)
Cytomegalovirus (CMV) infection	Ganciclovir (GCV)	GCV solid lipid nanoparticles containing borneol (GCVb-SLN) and GCV-SLN	KM mice	/	Injected by tail vein	Borneol-modified SLNs could increase the distribution of GCV to brain and AUC_0−T_ and C_max_ in mice liver and spleen	Loose intercellular tight junctions of the BBB	(62)
Ischemic stroke	Ginkgolides (GG)	Borneol-modified ginkgolides liposomes (GGB-LPs)	Male KM mice	Rat brain endothelia cells, bEnd.3	i.v. *via* the tail vein	Increased the uptake of bEND.3 cell, Cmax, AUC_0 → ∞_, MRT	Enhance blood-brain barrier permeability	(63)
**SKIN**								
The effect of S-BO on lymphatic targeting	7-ethyl-10-hydroxycamptothec	7-ethyl-10-hydroxycamptothecin liposomes (SN-38-Lips)	KM male mice	RAW264.7 cells of KM male mice	Subcutaneously injected	↑ Residence time, uptake of lymph nodes, intracellular and the medulla zone of PLNs fluorescent intensity, Cmax, t1/2, MRT0-24 h, CL, AUC0-24 h ↓ Injection-site retention not cause inflammatory reaction of injection site	Opening the barrier gap, accelerate the movement of biofilm	(64)
Gout	Colchicines	Borneol-physically-modified colchicine ethosome (COL-bpES); borneol-chemically-modified colchicine ethosome (COL-bcES).	Male Sprague–Dawley rats	HaCaT cell	Coated above the ankle skin	↑ Penetration; AUC (0–∞); AUC (0–t); C max ↓ Cytotoxicity; joint circumference; TNF-α PGE2	No description	(65)
Ischemic stroke	Tetram-ethylpyrazine (TMP) and borneol (BN)	TMP and BN microemulsion (TEM-BN-ME)	Male Sprague–Dawley rats; New Zealand rabbits	/	/	↑AUC 0-inf; C max; percutaneous absorption and brain distribution of TMP	ATP-binding cassette transporters, tight junction protein, enhancement of vasodilatory neurotransmitters	(66)
**GASTROINTESTINAL MUCOSA**								
Pancreatic carcinoma	9-nitrocamptothecin(9-NC)	9-NC-loaded PLGA nanoparticles (PLGA-NPs)	Male SD rats	/	Intragastric administration	Borneol prolonged the *T_*max*_*, increased the *C_*max*_*, improved bioavailability and enhanced intestinal absorption.	Loose tight junction	(67)

### Corneal

The cornea consists of six different layers, epithelium, Bowman's membrane, stroma, Dua's layer, Descemet's membrane, and endothelium, which build a barrier to the entry of the drug into the inner eye (68). Due to the existence of tight junctions, the corneal epithelial barrier limits the entry of drugs not only through the transcellular pathway, but also through the paracellular pathway, thus limiting their therapeutic effects (69). Borneol is widely used in ophthalmic preparations for treating ocular diseases. Borneol has also been shown to reduce the drag of phospholipids of the lipid bilayer in corneal epithelium to drug passage and loosen the tight junctions existing in the corneal epithelium, thereby allowing the paracellular transport of hydrophilic drugs and inducing drugs to pass through the cornea (70). Compared to other penetration enhancers, the major advantages of borneol are its wide use for ocular disease and long-term clinical safety.

The study demonstrated that 0.1% borneol increased corneal epithelial permeability to lipophilic and hydrophilic drugs *in vitro*, particularly hydrophilic drugs, and did not increase corneal hydration and damage the cornea and other ocular tissues. The mechanism by which borneol enhances hydrophilic drug permeation of the cornea by loosening the tight junctions between epithelial cells, which in turn, accelerate the passage of hydrophilic materials through the epithelium *via* the paracellular route (33). The corneal barrier consists of the epithelium, stroma, and endothelium, which form the three primary layers through which substances can permeate. The epithelium is the main barrier for water-soluble drugs, while the endothelium and stroma are the main barriers for many lipophilic drugs. Borneol, which is a lipophilic substance, may have some influence on the permeability of the stroma. Thus, borneol may have enhanced the cornea penetration of lipophilic puerarin, while it has little influence on the corneal penetration of hydrophilic timolol maleate (32). Li et al. demonstrated that borneol could enhance the diffusion of Danshensu across the ocular-blood barrier, but the detailed mechanism of its osmosis-promoting function needs to be investigated further (30). Additionally, borneol could loosen the blood-optic nerve barrier reversibly without causing damage by regulating the reversible translocation of two tight junction proteins (claudin-5 and occluding) between the cell membrane and the cytoplasm to improve the permeability of the blood-optic nerve barrier, while not changing the levels of claudin-5 or occluding (34). Menthol, combined with borneol, enhanced the *ex vivo* corneal permeability of fluconazole better than menthol alone. The possible mechanism may be that menthol has an alcoholic hydroxyl group, which is a hydrogen bond acceptor/donor. Furthermore, borneol could bind to the site on the cellular membrane or adsorb to the membrane surface to improve the membrane fluidity of the corneal epithelium, increase the orderly arrangement of cellular membrane phospholipid molecule chains, and reduce the collision with phospholipid molecules when fluconazole crosses the membrane, thereby facilitating drug permeation (35). Researchers found that borneol could increase rhodamine B, sodium-fluorescein, and FITC-dextrans of 4 kDa into the eye through the transcorneal pathways without causing ocular irritation and toxicity but failed to significantly corneal permeation of FITC-dextrans of 10, 20, and 40 kDa. The mechanism was that borneol quite possibly loosens the epithelium junctions in the cornea to an extent that is within a limit (50). Natural borneol enhanced the permeability and bioavailability of geniposide in the cornea, possibly because natural borneol is a small lipophilic compound and can be rapidly absorbed *via* the corneal tissue because of its liposolubility and low molecular weight. When passing through the corneal tissue, natural borneol might enhance epithelial junction permeability and promote paracellular drug absorption. In addition, no ocular damage or clinical abnormal signs were observed in the cornea, conjunctiva, or iris in ocular irritation tests (36) (Table 1).

Due to the existence of ocular blood-aqueous and blood-retinal physiological barriers, drug bioavailability (<5%) after ocular administration is often very low. The major approaches that have been explored to improve ocular drug bioavailability are drug delivery systems and penetration enhancers or prodrug approaches (71). Nanoparticles (NPs) have been shown to enhance precorneal retention and provide sustained drug delivery. But there seem to be no applications of borneol coadministration with drugs co-loaded with nanocarriers in the cornea. One study chooses five penetration enhancers having different modes of action, namely, benzalkonium chloride (BAC), capric acid (CA), Ethylenediaminetetraacetic acid (EDTA), sodium glycocholate (SG), and sodium taurocholate (ST) to explore their effect on nanoparticle system [polycaprolactone-pluronicF68 (PCL-PF68)] bioavailability to various ocular tissues. Results demonstrated that all the penetration enhancers enhanced the ocular permeability of PCL-PF68 nanoparticles to the anterior part of the eye except EDTA. BAC and CA increased bioavailability of PCL-PF68 nanoparticles in the conjunctiva, SG in the cornea, iris and ciliary body, and ST in the cornea. The mechanism was that SG and ST enhance drug delivery in the cornea by widening the tight junctions between cells, allowing penetration of drugs *via* the paracellular route, and affecting membrane fluidity (72). The above research shows that borneol can improve the membrane fluidity of the corneal epithelium, loosen the tight junctions between cells, and accelerate the passage of hydrophilic materials through the epithelium *via* the paracellular route (33, 50). Thus, the combination of borneol and surface properties of nanoparticles can differentially influence ocular permeability and bioavailability and can advantageously develop improved ocular drug delivery systems.

### Skin

The biggest challenge of the transdermal drug delivery system is that it is difficult for the drug to penetrate the highly organized structure of the stratum corneum, which is the most impermeable barrier to the skin (51, 73). On the other hand, the diffusion of drug molecules in the stratum corneum will be affected by its intercellular lipids, which present a high resistance to the penetration of molecules into the inner layer of the epidermis (74). Penetration enhancers change the structure or consistency of the stratum corneum and adjust the solubility and thermodynamic activity of the drug to promote its diffusion across the skin (75). Although the incorporation of suitable chemical permeation enhancers is a well-established strategy, it is easy to cause skin allergy and irritation. Borneol is a highly lipophilic, low molecular weight, volatile monoterpene, and is widely used as penetration enhancers in clinics with high safety, low toxicity, and side effects (51, 76). It was proposed that skin penetration enhancers may act by one or more of three potential mechanisms. Firstly, borneol can alter the intercellular lipid structure between the corneocytes to increase diffusivity (77). Secondly, borneol can dilate the subcutaneous capillaries so that the drug can easily enter the blood circulation and promote absorption. Thirdly, they may regulate the expression of P-gp, thereby affecting the transport function and ultimately promoting drug absorption (52, 78).

Borneol could markedly enhance the transdermal absorption of five model drugs, namely 5-fluorouracil, antipyrine, aspirin, salicylic acid, and ibuprofen. The IC50 values of borneol were markedly higher in both HaCaT keratinocytes and CCC-HSF-1 fibroblasts in comparison to those of the known standard enhancer Azone, a well-established and standard enhancer, indicating that borneol had relatively low toxicity on the skin cells. The molecular mechanism was that borneol could disrupt and extract part of the stratum corneum intercellular lipids, which would greatly perturb the orderly arrangement structure of the stratum corneum. In addition, borneol contributed negligibly to the alteration of the keratin conformation (51). Borneol has also been shown to reduce the collision and resistance between phospholipid molecules and increases its fluidity by changing the integrity of lipid cell membranes. On the other hand, there was a significant effect on the P-gp in the cell membrane, while no changes in the nucleus, thereby weakening drugs efflux and promoting the transdermal absorption (52). The comparative assessment indicated that borneol imparts a relatively stronger penetration-enhancement effect on 5-fluorouracil when compared to menthol. The mechanism was that borneol imparts a stronger disruptive effect on the morphology of the stratum corneum owing to the interaction between borneol and the head group of stratum corneum lipids is stronger than that of menthol. Furthermore, borneol-induced the duration of transient pore existence time longer than menthol, thereby increasing the diffusion coefficient of 5-fluorouracil (53) (Table 1).

Studies have been conducted to explore the potential of combining borneol with nanocarriers in transdermal drug delivery. Ye et al. (64) found that synthetic borneol (S-BO) enhanced the lymphatic-targeting ability of 7-ethyl-10-hydroxycamptothecin liposomes (SN-38-Lips) and reduced injection-site retention, prolonged residence time, and increased uptake of lymph nodes, which would not cause inflammatory reaction of the injection site. S-BO improved lymphatic uptake of SN-38-Lips *via* increasing cellular uptake of lymphocytes and macrophages in the lymph node, especially through phagocytosis of macrophages. SN-38-Lips did not show apparent change when were incubated with S-BO suspension for 12 h, which may relate to that S-BO decrease the entrapment efficiency *via* accelerating the movement of biofilm. The mechanism was associated with the ability of borneol to open the barrier gap. Zhang et al. used colchicine (COL) as a model drug, prepared borneol physically modified colchicine ethosome (COL-bpES), and borneol chemically modified colchicine ethosome (COL-bcES) by connecting borneol (BO) and dioleoyl phosphoethanolamine (DOPE) with succinic anhydride. The particle diameter of the COL-bcES was lower than that of the COL-bpES, and the toxicity, *in vitro* diffusion, pharmacokinetics, and pharmacodynamics were all superior to those of COL-bpES. It was related to the extreme lipophilicity of borneol that it encapsulated into the lipid core of COL-bcES easily. Borneol of COL-bcES had a more stable distribution in the surface of nanocarrier when ethosome passed through the skin, which makes borneol take better effect (65). Hu et al. found that borneol enhances the percutaneous absorption and distribution of tetramethylpyrazine (TMP) in the brain without adverse effects on the skin when TMP and borneol are combined and incorporated into a microemulsion-based transdermal therapeutic system. The mechanism may be consistent with the previous study, which included the modulation of multiple ATP-binding cassette transporters, tight junction protein, and the predominant enhancement of vasodilatory neurotransmitters mediated by borneol (66) (Table 2).

### Gastrointestinal and Nasal Mucosal

The inner wall of the gastrointestinal tract (GIT) is an important protective barrier as the presence of tight junctions prevents the entry of drugs through the paracellular pathway. At the same time, the ABC transporter that exists in the epithelial cells of the GI tract is involved in the efflux of various drugs back into the GI lumen, which also severely limits the permeability of drugs. Most nasal absorption enhancers, such as surfactants, overcome the transcellular barrier by disrupting the lipid structure of the cell membrane (56). EDTA facilitates paracellular transport by opening tight junctions (54, 79) and piperine promotes permeation of drugs by inhibiting the efflux pump (55, 80). The possible mechanisms of most nasal absorption enhancers include increasing membrane fluidity, reducing mucus viscosity or elasticity, inhibiting enzyme activity, inhibiting the efflux proteins, opening tight junctions, or dissolving drugs to enhance their diffusion (81). Borneol served as an effective penetration enhancer, which can play its penetration promotion role through most mechanisms described above.

Borneol promotes intestinal absorption, increases the tissue distribution, and inhibits the rapid metabolism of the notoginsenoside R1 and ginsenoside Rg1 and Re, possibly by loosening the intercellular tight junctions (54). Coincubation with borneol improved the apparent permeability coefficients (Papp) of curcumin (CUR) across the duodenum, jejunum, and ileum. The explicit reason was not described, just assumed that may be related to downregulation of ZO-1 and Factin associated with the tight junction, inhibition of the function and expression of P-gp, and multidrug resistance-associated proteins, which were important transporters found in the small intestine, alteration of the lipid phase of the intestinal mucous membrane, and acceleration of the fluidity of the polar head group regions in cell membranes according to the previous studies (55). It has been found that the nasal transport of geniposide increased significantly when co-administrated with borneol and muscone. The drag of phospholipids of the lipid bilayer in the nasal epithelium is the major barrier for topically applied drugs. The enhancing effect of borneol and muscone on geniposide transport might be linked to the combined effect of opening the tight junction proteins and increasing membrane fluidity. The possible mechanism may be that borneol has an alcoholic hydroxyl group, which is a hydrogen bond acceptor/donor (56) (Table 1).

Borneol plays the same role in increasing the intestinal epithelium permeability of NPs and chemical compounds. The results of the preclinical pharmacokinetic study showed that T_max_ (h) and C_max_ (ng/ml) values were significantly higher in nanoparticles (NPs) containing borneol (2.00 ± .81 h and 185.56 ± 27.78 ng/ml) as compared to 9-nitrocamptothecin (9-NC) solution (0.25 ± .77 h and 80.75 ± 22.85 ng/ml) and for NPs without borneol, it was 1.17 ± .41 h and 111.06 ± 18.87 ng/ml, respectively. The relative bioavailability of NPs containing borneol was 383.90 and 150% with respect to 9-NC solution and NPs without borneol, respectively. Poly lactic-co-glycolic acid NPs (PLGA-NPs) of 9-NC containing borneol only promoted intestinal transport for NPs with diameter <100 nm but not larger NPs. This may be explained by the ability of borneol to open tight junctions among intestinal epithelial cells and enhance the transport of smaller NPs through the paracellular pathway (67) (Table 2).

### Summary

These studies indicate that borneol exerts a regulatory effect on the BBB, cornea, skin, and mucous membrane permeability, which is considered as a strong penetration enhancer. The mechanism was generally interpreted as follows: Borneol could change the close connection between the epithelial cells (46, 50, 56) and accelerate the transportation of drugs through the intercellular passage (54). It could also increase the number and volume of pinocytosis vesicles in BBB cells (56), improve cell membrane permeability (36), change the integrity of the lipid cell membrane, and decrease the collision and the resistance between phospholipid molecules to increase their fluidity. Finally, borneol could significantly inhibit the activity of drug resistance proteins such as multidrug resistance mutation 1 (MDR1) and P-gp and accelerate the transportation of drugs (52). In addition, nanocarriers modified by borneol chemically show higher safety and better drug absorption properties than those modified physically. Therefore, it is necessary to review the effect of borneol co-modification with nanocarriers on the efficacy of antitumor.

## Recent Application of Nanocarriers Co-modified with Borneol for Solid Malignancies

**Figure 2:**
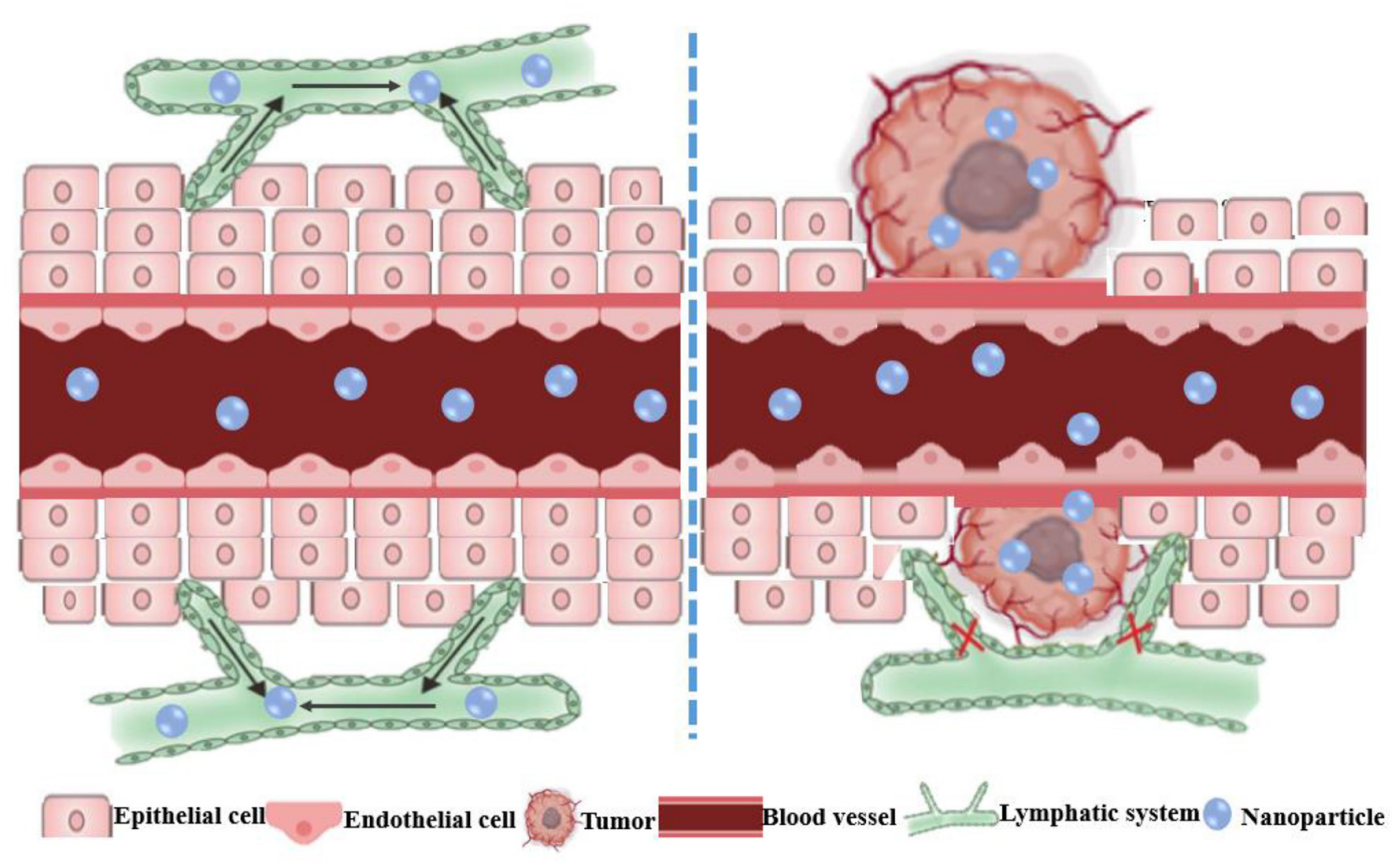
Schematic diagram of the enhanced permeability and retention (EPR) effect of nanoparticles in tumors. The normal tissue has tight vascular endothelial to prevent the extravasation of macromolecules. The EPR effect is the result of the leaky vasculature and ineffective lymphatic drainage of the newborn tumor blood vessels, which results in the retention of macromolecules in the tumor.

Nanoparticles integrate small drug molecules into nanomaterials through encapsulation or adsorption to achieve effective drug delivery (36, 82). NPs specifically accumulate in tumors due to passive targeting *via* the EPR effect and the increased pore diameter and hydraulic conductivity of the vasculature (Figure 2) (83). Importantly, the conjugation of different antitumor drugs to NPs can not only reduce the toxic side effects caused by large doses of single drugs, but also enhance its overall performance effect of cancer cells through multiple pathways, thereby reversing MDR (84–86). An NP diameter between 20 and 200 nm can increase drug uptake and passive targeting through the EPR effect (87, 88). Nanocarriers extend the circulation time of the drug in the body by adding some modifications, which can reduce phagocytosis by mononuclear macrophages and the reticuloendothelial (89). For example, some nanocarriers modifications such as folic acid (90), hyaluronic acid (91), cell membrane penetrating peptide (92), transferrin (93), and biotin (94) enhanced the targeting effect of chemotherapeutic drugs and increased the accumulation in the tumor site by binding to receptors that are specifically and highly expressed on the surface of tumor cells. In addition, many materials used for the modification of nanocarriers can reverse MDR, such as Poloxamer P85 (95), Poloxamer 188 (96), and polyethylene glycol vitamin E succinate (TPGS) (97).

Despite their promise, the efficiency of NPs delivered to tumor sites is low, with <0.7% of the total amount reaching the target site (21), which greatly hinders the clinical transformation. Based on the strong penetration property of borneol, the introduction of borneol into the nanocarriers is expected to increase the transmembrane absorption and the delivery efficiency into tumor sites. However, there are few studies in this area, especially those with antitumor applications. In this study, we review the recent studies on nanocarriers that have been co-modified with borneol, including NPs, nanoemulsions, liposomes, dendrimers, polymer micelles, and lipoprotein nanocomposites for improving therapeutic efficacy, reducing the toxicity, inhibiting tumor metastasis, reversing MDR, and enhancing brain targeting, to expand its application in targeted drug delivery systems in the treatment of solid tumors (Table 3).

**Table 3:** Borneol modified nanoparticle-based drug delivery platforms.

**Tumor type**	**Drug**	**Structure**	***In vitro***	***In vivo***	**Action results**	**Mechanism**	**References**
**NANOPARTICLES**							
Glioma	Paclitaxel	Borneol combined with CGKRK peptide modified paclitaxel prodrug self-assembled redox responsive nanoparticles (CGKRK-PSNPs)	U87MG cells, BCEC cells	U87MG glioma-bearing Balb/c nude mice, CGKRK-PSNPs injected *via* tail vein	↑ Cellular uptake of nanoparticles, cytoplasm uptake of PTX, proliferation inhibition, migration efficiency of both PEG-PSNPs and CGKRK-PSNPs penetrating the *in vitro* BBB, glioma site distribution of CGKRK-PSNPs combined with Bor, medium survival time of mice treated with CGKRK-PSNPs combined with Bor	Enhance BBB penetration	(98)
Hepatocellular carcinoma	Selenium nanoparticles (SeNPs)	Surface decoration of galactosamine (GAL) and Borneol (Bor)-modified SeNPs (GAL/Bor @ SeNPs)	R-HepG2 cells overexpressed P-gp; L02 normal hepatocyte	Sprague–Dawley (SD) mice	↑ Drug accumulation and retention, Cleaved-PARP, p-p53, p-ATM, p-BRCA1, P-Histone (Ser139), p-JNK, p-p38, MAPK ↓ABC family proteins, ROS, caspase-3/8/9, p-AKT, p-ERK	ABC family, p53 pathway, MAPKs and AKT pathway	(99)
Glioma	Ginsenoside-Rh2	Ginsenoside-Rh2 lipid nanoparticles	Glioma C6 cells	/	↓ Cell proliferation	/	(100)
NSCLC	Gefitinib	NBNPs	A549 cells	A549-bearing Balb/c nude mice	↑ ROS generation, DNA damage, cell apoptosis ↓ Cell proliferation, tumor weight, tumor volume, the injury of heart, liver and kidney	ROS, DNA damage, apoptosis	(101)
Lung, breast, cervical, malignant melanoma, liver, and colon cancer	doxorubicin (DOX)	PLGA@NB	A549, HepG2, SW480, MCF-7, A375 cells	A549-bearing Balb/c nude mice	↑ Drug accumulation, ROS generation, cell apoptosis ↓ IC50, tumor weight, tumor volume, the rate of tumor inhibition no heart, liver, and kidney injury	ROS, apoptosis	(102)
**NANOEMULSION**							
Glioma	*Brucea javanica* oil	Born4eol and *Brucea javanica* oil nanoemulsion	Rat glioma C6 cells	C6- bearing male Wistar rats	↑ The rate of tumor inhibition ↓ Tumor weight, tumor volume	Open the BBB, improved the brain targeting and drug distribution in the brain	(103, 104)
NSCLC	Gefitinib	Nanolization of NB (NBNPs)	A549, WI38 (normal lung fibroblast cells)	A549 tumor-bearing female Balb/c nude mice	↑ RIPK1, FADD mRNA and protein level; pharmacokinetic parameters (t_1/2β_, AUC_0−24*h*_, MRT); survival rate; NB concentration in the tumor region ↓ EHD1 mRNA and protein level; CI; tumor volume index, tumor volume, tumor weight; PCNA, Ki67	ROS generation, cell apoptosis, EHD1/FADD pathway	(105)
**LIPOSOMES**							
Refractory or recurrent brain tumors	Doxorubicin hydrochloride, l-borneol	Doxorubicin hydrochloride	/	Dox-NanoLips were injected by vein and borneol	↑ MRT, distribution phase rate constant (α), AUC_0−6h_, brain bioavailability and	Loose tight junction	(106)
		nanoliposome (Dox-NanoLips)		was intragastrically administered in mice	the brain–plasma ratio of Dox-nanoLips, the delivery of DoxnanoLips into the brain, DTP, DTE, the concentration of Dox in the left- and right-cerebral cortex and hippocampus ↓ t_1/2_, AUC_0−24*h*_		
The effect of S-BO on lymphatic targeting	Synthetic borneol (S-BO), 7-ethyl-10-hydroxycamptothec	7-ethyl-10-hydroxycamptothecin liposomes (SN-38-Lips)	RAW264.7 cells	Subcutaneously injected into the right footpad of KM male mice	↑ Residence time, uptake of lymph nodes, intracellular and the medulla zone of PLNs fluorescent intensity, C_max_, t_1/2_, MRT_0−24*h*_, CL, AUC_0−24*h*_ ↓Injection-site retention not cause inflammatory reaction of injection site	Open the barrier gap, accelerate the movement of biofilm	(64)
**DENDRIMERS**							
Glioma	Doxorubic (DOX)	A glioma targeted drug delivery system for DOX based on BO- and FA-dual-modified PAMAM (FA-BO-PAMAM/DOX)	Human Brain Microvascular Endothelial Cells (HBMEC) and C6 glioma cells	In-site xenograft glioma Model by Wistar rats	↑ Inhibition of C6 cells, BBB penetration, C6 cell uptake of DOX, circulating time, AUC_0−inf_, T_1/2β_, MRT, the AUC values in the brain and tumor, the tumor volume inhibitory ratio (%), body weight, rat survival, apoptotic cells in the tumor tissue ↓ The cytotoxicity of PAMAM, volume of distribution (VC), clearance (CL), cardiotoxicity	Enhance the BBB penetration	(107, 108)
Glioma	Doxorubic (DOX)	Borneol physical combination with doxorubicin (DOX) loaded PAMAM dendrimers drug delivery system modified with Angiopep-2 (ANG-PEG-PAMAM)	HBMEC and C6 glioma cell	/	↑ The transportation ratios for PEG-PAMAM dendrimers and ANG-PEG-PAMAM dendrimers, inhibition effect ↓ The release rates	BBB penetration	(109)
Ovarian cancer	Paclitaxel (PTX)	PTX and Borneol (BNL) co-loaded in the fabricatedPEG-PAMAM nanoparticle (NPs) (PB/NPs)	Paclitaxel-resistant ovarian cancer A2780/PTX cells	BALB/c nude mice bearing A2780/PTX cell xenografts	↑ Cellular uptake of PEG-PAMAM NPs, cytotoxicity, mitochondrial depolarization effects, PTX concentration in tumors, tumor necrosis ↓ Hemolysis rate, drug release, intracellular ATP level, P-gp, PTX concentration in liver, tumor growth, tumor size, Ki67	P-gp, Apoptosis, mitochondrial function	(110)
**POLYMERIC MICELLES**							
Non-small cell lung cancer (NSCLC), ovarian cancer	Paclitaxel (PTX), curcumin (CUR)	Polyethylene glycolpolynorbornene-thiocresol block copolymers (PEG-PNB-TC) loaded with PTX and CUR	Human non-small cell lung cancer A549, cervical cancer HeLa cells, multidrug resistant ovarian cancer cells (NCI/ADR-RES)	/	↑ Blood circulation, drug accumulation, cell uptake of the PEG-PNB-TC micelles, cell killing capability ↓ Drug release, drug clearance,	No description	(111)
Cerebrovascular and cerebral degenerative diseases	Vinpocetine	Vinpocetine loaded mixed micelles together with borneol	/	Male SD rats	↑ Micelle stability and compatibility, drug loading, drug release, the bioavailability and the mucosal absorption of VIN, drug distribution in the brain, brain targeting ↓Absolute bioavailability of VIN in plasma, Tmax,	Loose the intercellular tight junction; decrease ZO-1 and F-actin; increase the number and volume of pinocytosis vesicles in BBB cells; inhibit P-gp, Mdr1a and Mdr1b	(112)
Glioblastoma	Doxorubicin (DOX)	Conjugated borneol molecules with DSPE-PEG2000-COOH to synthesize a carrier DSPE-PEG2000-BO and loaded with DOX (DOX BO-PMs)	C6 and HBMEC cells	The *in situ* glioblastoma model build by ICR mice	↑ The cellular uptake of DOX-loaded nanomicelles, micelles' permeability, the transport ratio of DOX BO-PMs, anti-proliferation efficacy, the caspase-3 activity, TUNEL-positive cell ↓ IC50, C6 cell migration, the time to peak in brain, tumor volume, hemorrhaging and necrosis cell	Open the intercellular tight junction; cell apoptosis	(113)
Glioma	Carmustine (CMS)	Pep-1 and borneol -bifunctionalized carmustine-loaded micelles (Pep-1/Bor/CMS-M)	Human glioma BT325 and HBMECs cells	Orthotopic Luc-BT325 glioma tumor-bearing Balb/c (nu/nu) nude mice model	↑ The cellular uptake of micelles, the internalization of Pep-1/Bor/CMS-M, cell apoptosis, accumulation and the retention at the brain sites, survival period of mice ↓Cell proliferation, TEER values, tumor growth No obvious difference in body weight and spleen/liver index of mice	Apoptosis	(114)
**LIPOPROTEIN BASED NANOSCALE DRUG DELIVERY SYSTEMS**							
Glioma	Paclitaxel (PTX), borneol (BOR)	BOR and PTX co-encapsulated lipid-protein nanocomplex (BP-liprosome)	C6 glioma cells	Kunming mice bearing a C6 brain glioma xenograft	Exhibited a sustained release profile ↑The accumulation of BD-liprosome in the brain, the PTX concentration in the brain, the tumor inhibition rates, focal necrosis and nucleus pycnosis ↓Tumor weight, tumor volume	Apoptosis	(115)
Glioma	Paclitaxel (PTX), borneol (BOR)	Lipid-albumin nanoassemblies co-loaded with BOR and PTX (BOR/PTX LANs)	C6 glioma cells	Kunming mice bearing a C6 brain glioma xenograft	↑The cellular uptake of PTX, the uptake and the internalization of LANs, distribution of LANs in tumor ↓IC_50_, P-gp, ATP, liver toxicity	P-gp, clathrin- and endosome/lysosome-associated pathways	(116)
Cryptococcus neoformans meningitis and aspergillus brain abscess	Itraconazole (ITZ), borneol (BOR)	Bovine serum albumin nanoparticles (BSA-NPs) carried with ITZ and modified with both BO and PEG (PEG/BO-ITZ-NPs)	bEnd.3 cells	Male SD rats	**↑** The cellular uptake of nanoparticles, NPs' permeation through BBB, plasma level, t_1/2β_, mean residence time (MRT), AUC_0−t_, the distribution of nanoparticles in brain **↓** The release of ITZ, clearance (CL)	Clathrin-mediated pathway, improved BBB penetration efficiency	(117)

### Improved Therapeutic Efficacy

A previous study reported that borneol increased the transdermal absorption of drugs toward different barriers, which effectively increase the blood concentration and bioavailability of other drugs. Borneol is always used as an adjuvant in combination with other drugs to reduce the dosage of other drugs, increase their therapeutic effect, and decrease drug side effects (118). In recent years, many studies have shown that borneol often served as an antitumor chemotherapeutic sensitizer works along with the chemotherapeutic drugs to promote anticancer effect by increasing the level of reactive oxygen species (ROS) (119), arresting cell cycle (120), and regulating the expression of proapoptotic MAPK family member proteins and PI3K/Akt pathway proteins (121). However, the volatility of borneol makes it extremely unstable during preparation and storage. In addition, the poor water solubility of NB is not conducive to blood circulation, which greatly limits the effective delivery to the treatment site and greatly reduces its therapeutic effect. The progress of nanotechnology in the field of biomedicine undoubtedly provides a novel method for expanding and improving the medicinal value of natural borneol (122). Nanotechnology has unique advantages in improving the stability, biocompatibility, and absorption of drugs, and is increasingly being used to design new delivery systems for drugs. Therefore, the introduction of borneol into the nanocarrier is expected to increase the transmembrane absorption and the delivery of drugs into tumor cells.

**Figure 3:**
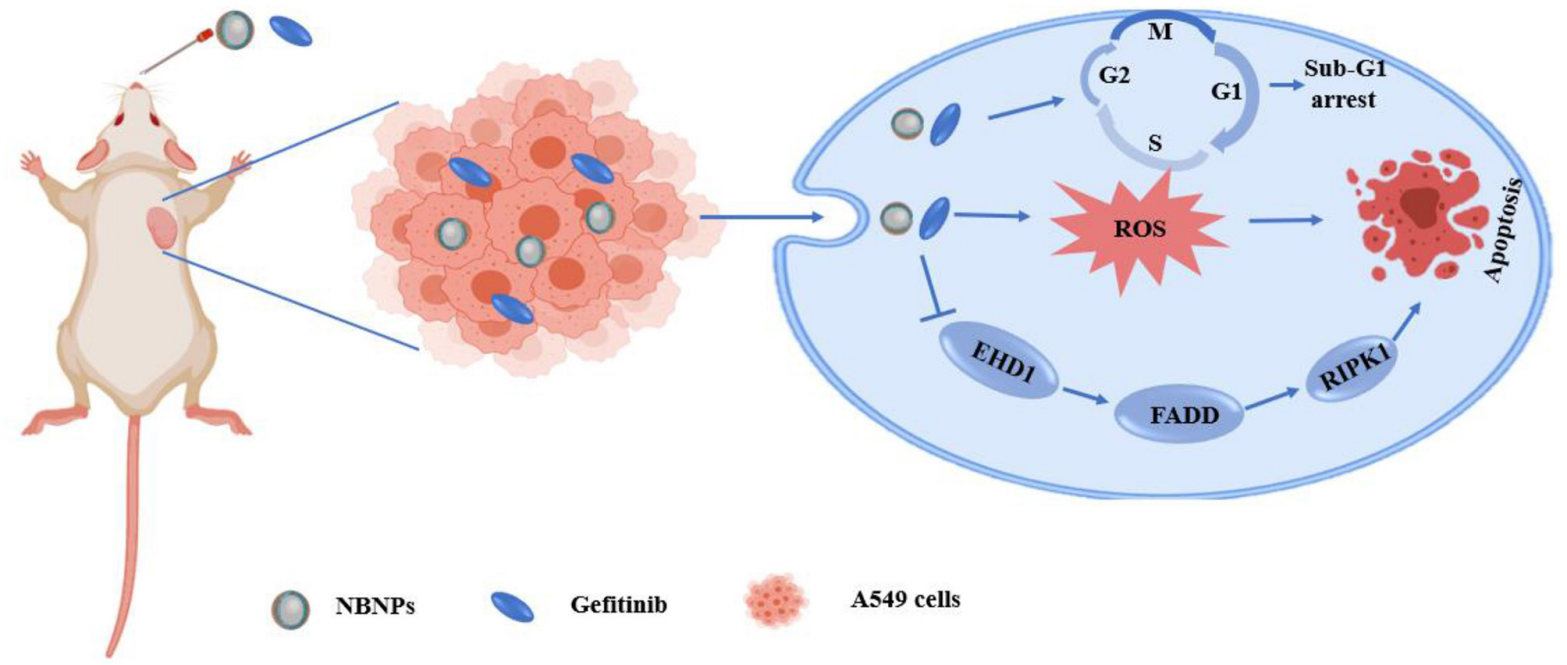
Schematic illustration of the mechanism of the natural borneol nanoparticles (NBNPs) enhanced gefitinib sensitivity in A549 cells when loaded with natural borneol.

Yuan et al. (105) used Tween 80 as surfactant, olive oil as an oil phase, and incorporated natural borneol into the hydrophobic core of NPs so that the hydrophilic phase extended outward into the hydrophilic environment. These alterations greatly improved the water solubility of natural borneol and prevented the volatilization of the NPs during the drug delivery, which induced NB crystals were easy to be dispersed in the nano system of natural borneol NPs (NBNPs) after nanolization. MTT results indicated that the inhibition efficiency of NBNPs + gefitinib (GFT) was considerably higher than that of free NBNPs and free GFT. The combination indices (CI) value suggesting that the NBNPs+ GFT (0.931) group exhibits a synergistic effect, while the NB + GFT (1.046) group shows a slight additive effect. In addition, NBNPs exerted excellent selectivity between A549 and normal lung fibroblast cells WI38 cells, exhibiting great targeting. These results demonstrated that nanolization of NB improved the antitumor activity and sensitization to GFT than free NB, owing to how some moieties of NBNPs had been exposed after nanolization and then enhanced the cellular uptake of GFT. The mechanisms of NBNPs enhanced GFT therapy in A549 cells by arresting the cell cycle in the Sub-G1 phase, increasing ROS, suppressing DNA synthesis, and promoting cell apoptosis. Proteomics results revealed that further mechanism was NBNPs inhibiting EGFR-associated protein EHD1 to cascade apoptosis-associated signaling pathway (Figure 3). In another study, Han et al. (109) fabricated a poly amido amine (PAMAM) dendrimer loaded with doxorubicin (DOX) modified with Angiopep-2 (ANG/PAMNA/DOX) because DOX cannot pass through the BBB in the body. Angiopep-2 modification could enhance the affinity between the dendrimers and finally increase the cell uptake and boost the antitumor ability. The result showed that borneol co-incubation significantly enhanced the transportation ratios of ANG/PAMNA/DOX mainly caused by the promotion effect of borneol for opening tight junctions of the BBB. Moreover, the self-assembled bottlebrush polyethylene glycol-polynorbornene-thiocresol block copolymers (PEG-PNB-TC) with paclitaxel (PTX) coloaded into their hydrophobic core could be efficiently taken up by cells *in vitro* and induced comparable cytotoxicity to the cancer cells. Compared with commercial PTX formulation (Taxol), the PTX-loaded PEG-PNB-TC micelles enhanced *in vitro* cellular uptake and comparable cytotoxicity, which is related to PEG-PNB-TC micelles that could be efficiently internalized by cancer cells most likely as an intact entity. PEG-PNB-TC polymers were safer and more biocompatible than Cremophor EL, which is the surfactant used in Taxol. In addition, PEG-PNB-TC micelles that could coload curcumin showed a synergistic effect to overcome the drug resistance at a high ratio of Cur/PTX, whereas the lower Cru/PTX ratio showed an additive effect. It provides a strategy for current cancer treatments in two ways: (i) ensuring to adjust multiple drugs with different ratios and higher drug loading; and (ii) the clinically used or well-investigated combination drugs could be directly applied to the PEG-PNB-TC micelles without further adjustment of drug ratios (111). Zou et al., finding that ginsenoside Rh2 lipid nanoparticles with the assistance of borneol has better effects with increased the solubility of ginsenoside Rh2, slowed the drug release, reduced the IC50 of C6 cells in a concentration-dependent manner, and improved the antitumor effect. The oral bioavailability of ginsenoside Rh2 increased after being loaded into lipid nanoparticles by increasing the residence time through the adhesion of the lipid layer and contact area. The specific antitumor mechanism is not described (100). It has been reported that lipid-albumin nano assemblies coloaded with borneol and paclitaxel (BOR/PTX LANs) enhanced the cytotoxicity and reversed MDR in C6 glioma cells. The BOR/PTX LANs displayed higher cytotoxicity against C6 glioma cells. However, the addition of free cyclosporine A (CsA) or BOR along with BOR/PTX LANs did not significantly increase PTX uptake, indicating that BOR/PTX LANS had sufficient ability for PTX to inhibit P-gp efflux. It was proved that the cellular uptake of BOR/PTX LANs by C6 cells was contributed for two reasons. First, the BOR/PTX LANs intracellular released BOR inhibits P-gp efflux. Second, endocytosis of BOR/ PTX LANs consumed ATP, which could reduce the activity of P-gp. Finally, the composition of BOR/PTX LANS was like the lipids and proteins of the cell membrane, which may have a higher affinity with the cell membrane of tumor cells to increase the cellular uptake, thus improving cell cytotoxicity. Additionally, the distribution of drugs in the liver was decreased, which weakened the side effects of drugs (116).

### Reduced the Toxicity

Yuan et al. (105) found that NBNPs + GFT treatment not only exhibits a higher tumor inhibitory effect than NB + GFT or GFT treatment, but also showed a higher survival rate. The survival rate of NB + GFT and GFT groups was 85.9 and 75.0%, respectively, and 100% in the NBNPs + GFT group without significant body weight loss. The histopathological analysis indicated that the NBNPs + GFT group without evident pathological changes were caused in the heart, liver, spleen, lung, and kidney owing to the accumulation of NBNPs in the liver, spleen, and lung was gradually decreased as time went on to 24 h. In addition, the blood biochemistry indices effectively recovered the hematological indices, reflecting the physiological function of the kidney, liver, and lipid metabolism including blood urea nitrogen (BUN), aspartate aminotransferase (AST), total protein (TP), globulin (GLB), albumin/globulin ratio (A/G), and high-density lipoprotein cholesterol (HDL-C) at the normal level. These indices of the tumor-bearing mice have been changed to vary degrees compared to the healthy nude mice without tumor. These findings suggested that the combination therapy alleviated the tumor-associated toxicity and recovered the function of the major organs. Thus, NBNPs combined with GFT enhanced antitumor effect against NSCLC and without causing evident systemic toxicity. Effective targeting drug delivery system for glioma treatment is still greatly challenged by the existence of the BBB and the intracranial overspreading of the antitumor drug. If the drug reaches the non-glioma site after entering the brain, the concentration of the drug in the glioma site will decrease, and it may also cause more serious central system side effects. Poly amido amine (PHAMA) is an excellent carrier for brain drug delivery systems, which is beneficial to drug delivery and structural modification. But PAMAM with a positive charge usually has hemolytic and cytotoxicity (123). In previous studies, biocompatible polyethylene glycol (PEG) or lauroyl chloride is usually modified into a PAMAM dendrimer to reduce the toxicity induced by naked amine termination. However, these single modified vectors cannot effectively penetrate the BBB (124). A dual-functional glioma targeting delivery of doxorubicin was designed by the poly amido amine (PAMAM G5) dendrimer, modified with folic acid (FA) and borneol (BO) (FA-BO-PAMAM/DOX). Borneol modification reduced the potential toxicity of the PAMAM surface by reducing the amino groups on the surface of PAMAM. Furthermore, borneol modification increased the transport across the BBB ratio by 2-fold. Compared with the DOX alone, FA-BO-PAMAM/DOX increased the accumulation of DOX in tumor tissue, which means decreasing the drug in other sites. The fluctuation in animal body weight can reflect the side effects of drugs. The body weight of rats showed a slight increase at the early stage of tumor growth, compared with DOX alone. The cardiac toxicity was reduced because FA-BO-PAMAM/DOX effectively delivered DOX to the target site and decreased its content in the heart. At the same time, the median survival time of xenograft rats (28 days) was significantly prolonged compared to free DOX (18 days). Thus, modification with borneol not only decreased the cytotoxicity of PAMAM itself, but also facilitate DOX passage through the BBB easily (107, 108). Carmustine (CMS) is a commonly used drug for clinical treatment of glioma, with a short half-life of 15–30 min and lack of selectivity for tumor cells resulting in blood toxicity, bone marrow suppression, liver, and kidney injury. Reports suggest that borneol as a BBB permeation enhancer effectively improves the intracerebral delivery of nanodrugs. Guo et al. (114) developed Pep-1 and borneol-biofunctionalized CMS-loaded micelles (Pep-1/Bor/CMS-M), which is capable of penetrating the BBB and targeting glioma cells actively, thereby circumventing its limitations. Compared to CMS-M, Bor/CMS-M entered the cells more easily, which is probably related to how borneol modification reduced the integrity of the junction between endothelial cells, thereby facilitating the entry of particles into the BBB. Moreover, Pep-1/Bor/CMS-M did not lead to a significant decrease in body weight after five injections and obvious difference in the spleen/liver index and liver kidney function, exhibiting the longest survival period and low systemic toxicity in treating orthotopic glioma tumor-bearing nude mice. Natural borneol nanoparticles (NBNPs) combined with GFT not only inhibited the tumor growth, but also alleviated the heart, liver, and kidney damage and exhibited no obvious toxicity *in vivo* (101, 102).

### Inhibited Tumor Metastasis

Approximately 0.5% of all glioblastoma patients suffer extracranial metastases. Meng et al. (113) designed a drug delivery system borneol-modified DSPE-PEG2000-BO loaded with doxorubicin (DOX-BO-PMs). The results demonstrated that no obvious metastases occurred in the lung tissues. In addition, vascular infiltration was found obviously in the DOX solution and DOX PMs groups, whereas no abnormalities were observed in the tissues in the DOX-BO-PMs group. On the other hand, the DOX solution and DOX PMs groups found a significant liver metastasis, while no liver metastasis was observed in the DOX BO-PMs group. These results indicated that DOX BO-PMs exhibited a significant suppressive effect on tumor metastasis. In addition, DOX-BO-PMs enhanced the antitumor effect of DOX by inhibiting cell migration, increasing caspase3, and inducing cell apoptosis effectively. Furthermore, DOX-BO-PMs group did not show remarkable side effects on body weight maintenance of tumor-bearing mice. DOX-BO-PMs significantly enhanced the transport efficiency of DOX across the BBB and exhibited a quick accumulation in the brain tissues to enhance the antitumor effect of DOX. Although the researchers did not explain the mechanism of antimetastatic efficacy of DOX-BO-PMs, we speculate that the effect of inhibiting metastasis is related to its enhanced antitumor effect.

### Sensitizing and Reversing the MDR

In recent years, nanotechnology-based chemotherapy has exerted potential in cancer treatment. However, MDR limits the efficacy of chemotherapy. Nanocarriers, such as liposomes, lipid nanoparticles, polymeric micelles, and microemulsions have gained much attention to overcome MDR. Although they can improve the solubility of drugs and act as the intracellular drug reservoirs after entering the tumor cells (125). However, most of the free drugs were still expelled by the P-gp in tumor cells (126). Therefore, it is effective to overcome MDR of the tumor cells by co-encapsulation of the P-gp inhibitors and the anti-tumor drugs using a nanodrug delivery system. In addition, P-gp has been an important mechanism of multidrug resistance. It has been reported that overexpression of P-gp in tumor cells decreases the intracellular accumulation of DOX, paclitaxel, and cisplatin, which results in lower therapeutic efficacy. Therefore, suppressing P-gp expression has become an effective way to enhance chemotherapy efficacy and reverse chemoresistance (127). Borneol is bicyclic monoterpenoid alcohol with an antibacterial and anti-inflammatory effect. Furthermore, previous studies confirmed the role of penetration enhancer to improve oral bioavailability and increase the drug distribution in some tissues (37–39). Particularly, borneol has been reportedly used as a biologically active natural product in cell signal transduction, such as elevate the level of ROS, arrest the cell cycle, and regulate the apoptosis signal pathway (101). Furthermore, borneol can participate in reversing multidrug resistance by inhibiting the expression of P-gp (128). Therefore, the use of borneol to enhance membrane permeability and inhibit the expression of efflux proteins to design new nanocarriers loaded with chemotherapeutics may play an unexpected role in overcoming multidrug resistance.

Zeng et al. (99) fabricated novel cancer-targeted drug carrier selenium NPs (SeNPs) combined with borneol and galactosamine (GAL) (GAL/Bor@SeNPs). Borneol-modified SeNPs can significantly improve the stability of SeNPs and their anticancer activity. Nanoparticles with a diameter of <200 nm are more likely to enter the cell and increase the selectivity of the drug in the cancer cell. The use of borneol can significantly reduce the particle diameter of SeNPs. MTT assay results showed that GAL/Bor@SeNPs reduced the toxicity on L02 cells and improved the activity of drug-resistant hepatocellular carcinoma cells (R-HepG2) cells. GAL/Bor@SeNPs inhibited the expression levels of ABC family proteins (ABCB1, ABCC1, and ABCG2) in R-HepG2 cells in a dose-dependent manner, is consistent with borneol could participate in reversing multidrug resistance by inhibiting the expression of P-gp. The further mechanism was that GAL and borneol provide a rational strategy for the construction of a functional nanosystem to reverse multidrug-resistant cancers by inhibiting the expression of ATP-binding cassette family proteins, inducing cell apoptosis through ROS-activated p53 phosphorylation, mitogen-activated protein kinase (MAPK), and protein kinase B (AKT) pathways. The disadvantages of poor water solubility and unstable distribution of borneol in the body limit its application as a sensitizer. Cheng et al. (101) prepared natural borneol into nanoparticles (NBNPs) with borneol to prevent sublimation, thereby improving the stability of borneol. Compared with NB, NBNPs inhibited A549 cell proliferation by increasing the levels of ROS and DNA damage leading to cell apoptosis. Moreover, NBNPs enhanced the sensitivity of GFT by increasing the level of ROS and DNA damage, arresting the sub-G1 phase, leading to cell apoptosis furtherly. In another study, the PLGA loaded with d-borneol NPs (PLGA@NB) were administrated with doxorubicin (DOX) to A549 cells. The results revealed that PLGA@NB+DOX decrease the IC50 from 0.8 to 0.08 μm, and the antitumor activity increased by 10-fold. Moreover, the PLGA@NB+DOX improved the cellular uptake by 1.5-fold, increased the level of ROS to 169%, and remained above 165% within 2 h. The mechanism of enhanced the sensitivity of DOX was PLGA@NB+DOX elevating the ROS level and inducing cell apoptosis, which was consistent with the previous study (102). Zou et al. (110) coloaded paclitaxel (PTX) and borneol (BNL), a natural compound with P-gp inhibition effect confirmed in intestinal absorption, in fabricated PEG-PAMAM NPs (PB/NPs) using a one-step nanoprecipitation method and reserved the MDR of paclitaxel-resistant ovarian cancer A2780/PTX cells *in vitro* and *in vivo*. The combination of BNL could remarkably increase PTX cellular uptake in A2780/PTX cells. BNL was liable to bind with P-gp, confirmed by molecular docking. The docking calculation showed that BNL and verapamil were docked in P-gp molecule with a similar binding domain, in which most binding sites of the two drugs had a great deal of overlap. The potential MDR reversal mechanisms were co-delivery of PTX and BNL by PEG-PAMAM NPs would inhibit P-gp expression, enhanced mitochondrial depolarization effects, inhibiting intracellular ATP production to interfere with the mitochondrial function, promote cell apoptosis.

### Enhanced Targeting

The BBB acts as a physical barrier to prevent most chemotherapeutic drugs from crossing into the brain, which restricts antitumor effects. Borneol is a highly lipid-soluble bicyclic terpene, which can facilitate the distribution of central nervous system drugs in the brain *via* opening BBB. Previous studies have confirmed the mechanism involved in open the intercellular tight junctions, inhibit the P-gp, enhance pinocytosis in the BBB cells and improve the permeation of drugs across the BBB (44, 45). In the past few decades, targeted nanodrug delivery systems, such as nanoparticle, liposome, and dendrimer, have revolutionized the diagnosis and treatment of brain tumors. These nanocarriers still have some limitations, such as the low drug loading, premature release of the drug into the blood circulation, without active targeting, and poor permeability to cross the BBB (129). In addition, simple nanocarriers are quickly swallowed by macrophages in the reticuloendothelial system after intravenous injection, and many of them are targeted to the liver and spleen, leading to low brain targeting efficiency (130). Based on the above advantages of borneol, the modification of borneol in nanocarrier has great potential to enhance brain targeting.

**Figure 4:**
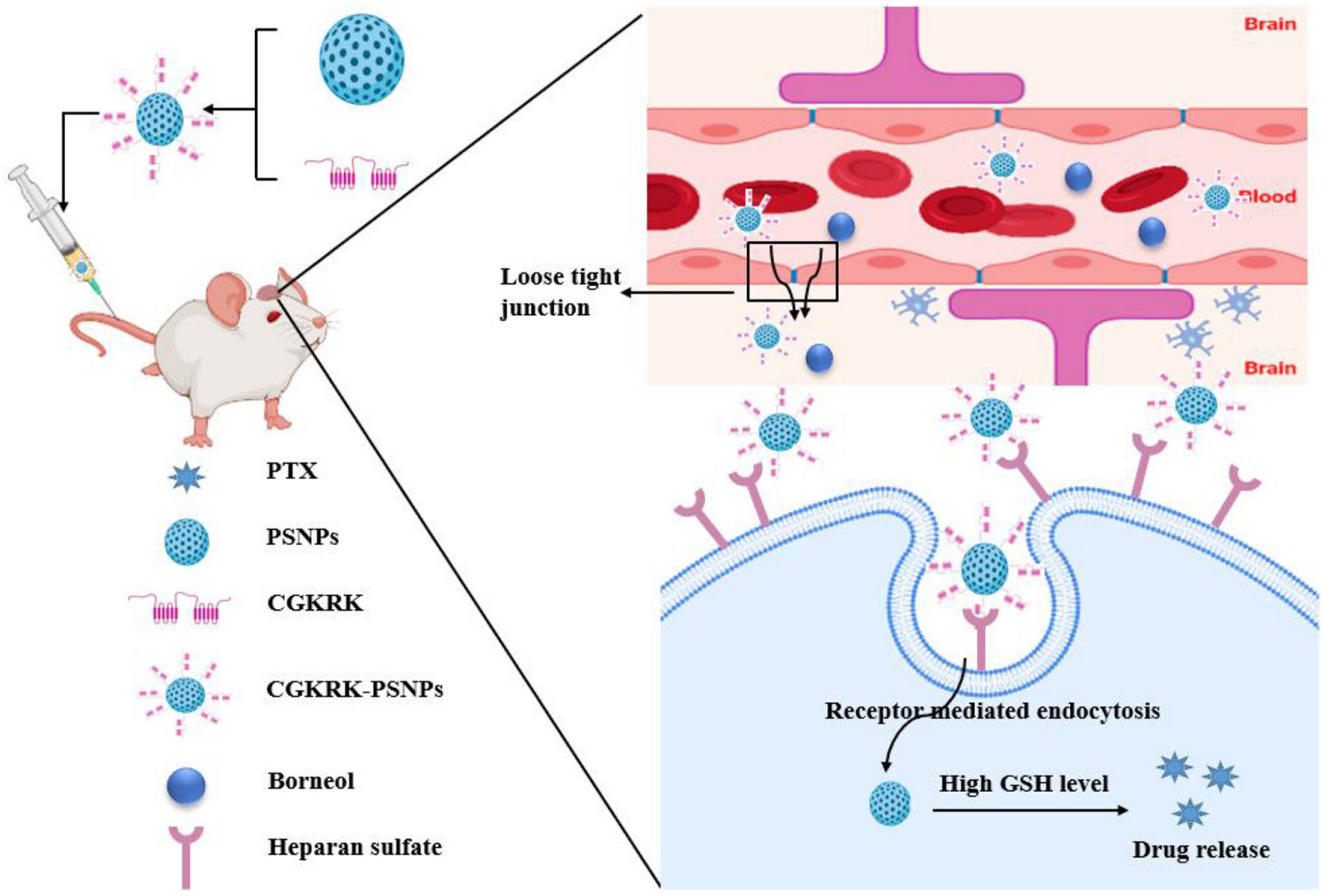
Schematic illustration of the application of the borneol combined CGKRK peptide modified paclitaxel prodrug self-assembled redox-responsive nanoparticles (CGKRK-PSNPs) for brain targeting in glioma treatment.

**Figure 5:**
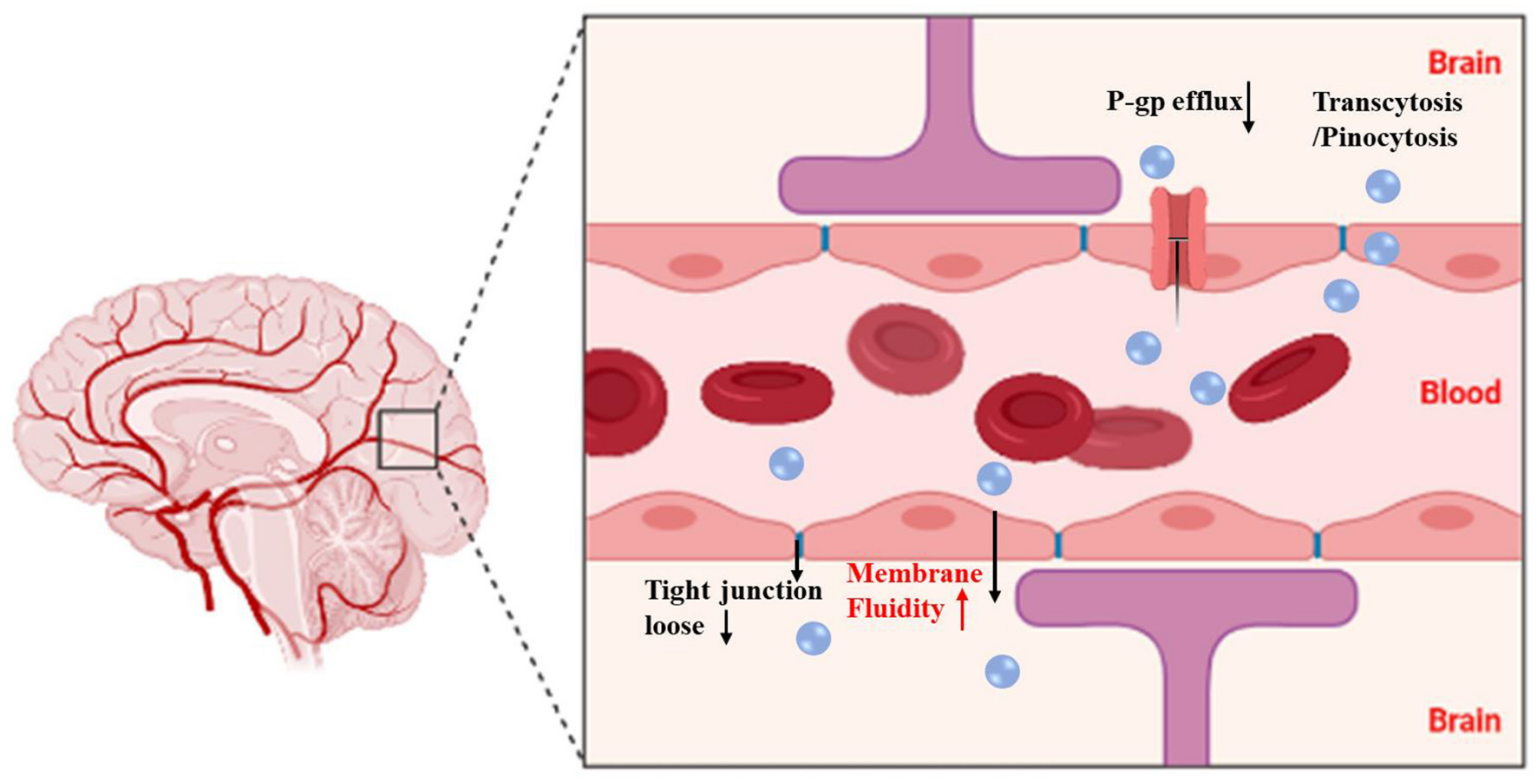
Schematic illustration of BP-liposome transport of paclitaxel (PTX) across the blood-brain barrier (BBB) and improvement of the anti-glioma effect of PTX. The figure was created by BioRender, https://app.biorender.com/.

The poor BBB penetration and low accumulation of therapeutic drugs at tumor sites are the major obstacles to achieving success glioma treatment. A previous study (98) investigated the efficiency of CGKRK peptide (a ligand of the heparan sulfate, which is overexpressed in glioma cells) modified paclitaxel prodrug self-assembled redox-responsive NPs (CGKRK-PSNPs) combined with borneol in glioma treatment. CGKRK was proved could target tumor cells and tumor neovascular through binding to the specific receptor heparan sulfate. PSNPs [paclitaxel prodrug (PTX-SS-C18) conjugate self-assembled nanoparticles] was used to accurately trigger-release PTX in tumor cells by the intracellular GSH. Borneol was intragastrically administrated for 0.5 h in advance to open the tight junctions of the BBB reversibly, and then CGKRK-PSNPs were injected *via* the tail vein and penetrated across the BBB to accumulate at tumor sites with the assistance of borneol active glioma target ability. The penetration efficiency of CGKRK-PSNPs with the assistance of borneol increased to 23.85% compared to without borneol (18.38%) *in vitro* BBB model established through BCEC cells. The images of *ex vivo* brains and the semiquantitative results also demonstrated that CGKRK-PSNPs combined with borneol exhibited the highest accumulation than other groups. These findings suggested that CGKRK-PSNPs significantly enhance the active tumor targeting efficiency owing to the CGKRK peptide-mediated endocytosis and BBB penetration ability of borneol (Figure 4). *Brucea javanica* oil emulsion is used to treat brain glioma by injection. The emulsion is a multiphase kinetic unstable dispersion system with a large diameter, easy demulsification and delamination, and a low concentration cross into the brain limits its clinical application. Li and Lv et al. (103, 104) successfully prepared borneol and *Brucea javanica* oil nano emulsion (BBNE) with a small particle diameter, high stability, and large drug loading. Results indicating that BBNE not only improved the stability of the drug, but also inhibited tumor growth. The mechanism may be related to borneol promoting the transport of drugs across to the BBB, increasing drug brain targeting, and improving drug distribution in the tumor tissue. Wu et al. (106) fabricated doxorubicin hydrochloride nanoliposomes (Dox-Nanolips). Co-administrated with borneol quickly enhanced the distribution of Dox-nanoLips to other tissues or organs with the increased value of the distribution phase rate constant (α), indicating that borneol may be beneficial to promote the brain targeting of Dox-nanoLips. Borneol increased the Dox concentrations of the Dox-nanoLips group in the cerebral cortex and hippocampus, while no change in the distribution of left and right-brain regions, indicating borneol has a specific targeting effect on Dox-nanoLips entering the cortex and hippocampus. The stronger red fluorescent signal of the borneol co-administration group was observed in the cerebral cortex, CA3, and dentate gyrus (DG) of the hippocampal region compared with Dox-nanoLips group, the GFAP (indicated astrocytes) fluorescent mainly appeared in the DG region, but the red fluorescent of Dox in the borneol co-administration group did not co-localize with the astrocytes in the merge images of the DG region. Thus, compared with Dox-nanoLips alone, Dox-nanoLips co-administer with borneol can directly across the BBB into the brain parenchyma by borneol opening the BBB tight junction, and then taken up into the neuronal cells bypassing astrocyte, which was beneficial to the higher neuronal targeting in the cerebral cortex, CA3 and DG region of the hippocampus. PTX cannot transport across the BBB and reach therapeutic concentrations in the brain tissue. Although the lipid-protein nanocomplex (liposome) was successfully prepared from egg yolk lecithin (PL 100M) and bovine serum albumin (BSA), the PTX transport across the BBB enhanced and systemic toxicity reduced compared to that of PTX solution. However, the brain tissue accumulation of PTX was still relatively low. Encouraged by the high potential of borneol as a promoter for drug transport across the BBB, a borneol (BOR) and paclitaxel (PTX) encapsulated lipid-protein nanocomplex (BP-liposome) was prepared. The cumulative release of PTX was lower than that of BOR in BP-liposome at all time points, which induce the earlier release of BOR to improve the permeability of the BBB, and subsequently increase the transport of PTX to the brain. BP-liposome enhanced the brain targeting of PTX by increased the accumulation of PTX in the brain. This is probably due to the particle characteristics of the BP-liposome and the synergistic effect of BOR. First, borneol accelerated the transport of drugs through intercellular channels by reducing the level of tight junction proteins in the intercellular junctions. Second, borneol could be bound to a site on the cell membrane or be absorbed to the membrane surface to improve the membrane fluidity of the epithelium. Consequently, the orderly arrangement of membrane phospholipid molecular chains was increased and the collision of drugs with phospholipid molecules was reduced, which promoted drug penetration through the cell membrane. Third, borneol inhibited P-gp expression, which inhibited drug efflux and enhanced the transport of drugs across the BBB (115) (Figure 5). Bovine serum albumin nanoparticles (BSA-NPs) served as one of the most appropriate carriers for drug delivery since it is biocompatible, biodegradable, non-toxic, and non-immunogenic. Based on this, a brain targeting drug delivery system for carrying ITZ was designed based on BSA-NPs, modified with both borneol (BO) and PEG (PEG/BO-ITZ-NPs). Modification of BO endowed ITZ-NPs with the brain targeting attributes, resulting in an exacerbated quantity of ITZ in the brain, this is attributed to borneol modification regulated membrane permeability, improved the mobility of phospholipid molecules, and inhibited P-gp mediated efflux, thereby increasing BBB penetration efficiency to enhance brain targeting and extend the retention time of the drug. Thus, BO and PEG dual modified BSA nanoparticles may potentially serve as an ITZ vehicle for brain targeting (117). The BBB represents a huge obstacle for the therapy of central nervous system related diseases. Polymeric micelles have many advantages for brain targeting by oral delivery, but the stability and targeting efficiency needs to be improved. How to bypass the BBB barrier *via* the oral route to achieve better brain targeting is a problem that must be solved. Ding et al. (112) investigated whether the oral absorption and brain distribution of vinpocetine (VIN) could be significantly improved by P123-based mixed micelles in combination with borneol. Compared to the micelles only group, VIN was absorbed faster after co-administration with borneol, indicating borneol enhanced brain targeting effect of VIN. In addition, the brain Ce [(C_max_)_brain_/(C_max_)_plasma_] was increased after peroral administration of P123 based mixed micelle, which enhanced passive brain-targeting. Taken together, the possible mechanism of enhanced peroral absorption and brain targeting of the VIN was the combination of the P123 passive targeting effect based on mixed micelles and the active targeting of borneol. The lymphatic system has become an important target and drug delivery route for preferential therapeutic. Ye et al. (64) found that synthetic borneol (S-BO) enhanced the lymphatic targeting of 7-ethyl-10-hydroxycamptothecin liposomes (SN-38-Lips) in the paracortex and medulla of the popliteal lymph node (PLN) cytoplasmic *via* increasing cellular uptake of lymphocyte and macrophages in the lymph node, especially through phagocytosis of macrophages.

### Summary

The use of nanocarriers modified by non-toxic and efficient natural products to enhance the therapy efficiency of traditional antitumor drugs is an innovative strategy in recent years. The above results revealed that borneol modification increased the cellular uptake of co-loaded drugs by inhibiting the efflux protein P-gp and then boosting the antitumor effect by arresting the cell cycle, increasing ROS, suppressing DNA synthesis, and promoting cell apoptosis. Borneol modification can reduce the toxicity of some nanocarrier material like PAMAM and increase the accumulation of drugs in the tumor site, thereby reducing the toxicity of other organs, such as the heart and liver, and prolong survival time. The above results also revealed that borneol modified exhibited a better multidrug resistance reversal effect, the possible involved mechanisms including increasing the accumulation of drugs in tumor site by inhibiting P-gp induces drug efflux, blocking the cell cycle, increasing the level of ROS, and promoting cell apoptosis. Finally, borneol modification enhances the targeting, especially in the brain. Borneol served as a promoter for drug transport across the BBB by loosening the tight junction, improving the membrane fluidity of the epithelium, and inhibiting P-gp mediated drug efflux. Furthermore, nanolization of borneol not only improves its water solubility, but also prevents volatilization loss during the administration process, overcomes poor drug stability, and exhibits stronger targeting and cytotoxicity. In addition, the modification of the small molecule borneol also simplifies the synthesis process and reduces the synthesis cost. Therefore, borneol has potential advantages in the construction of candidate nanocarriers material to enhance the targeting effect, overcome multidrug resistance, and reduce the toxicity of drugs or nanocarrier material.

## Conclusions

**Figure 6:**
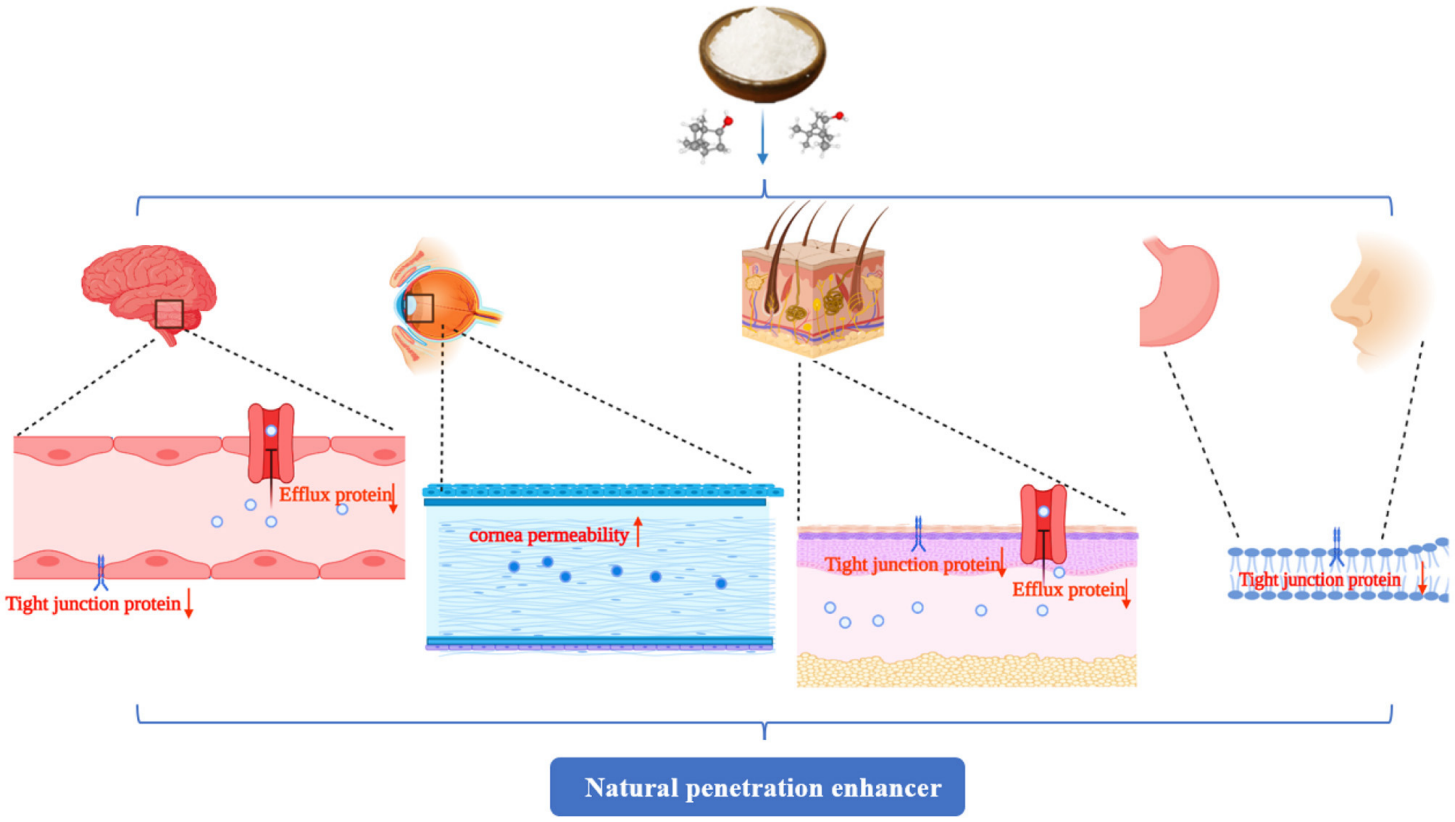
The penetration promoting effect of borneol is mainly reflected in promoting drug penetration through the skin, gastrointestinal mucosa, nasal mucosa, corneal, and the blood-brain barrier (BBB). The main mechanisms involved changes in the arrangement of lipid molecules and increases in their fluidity, improved mucosal cell permeability, increased paracellular and intercellular transport, destruction of the tight junction proteins, opened the BBB, and inhibited the expression of drug-resistant proteins, such as multidrug resistance mutation 1 (MDR1) and P-glycoprotein (P-gp), to reduce the drug efflux. The figure was created by BioRender, https://app.biorender.com/.

Borneol is a courier herb that has been used for thousands of years to facilitate the transport of co-administered drugs. The mechanisms of borneol as a permeation enhancer for delivering drugs across the BBB, corneal, skin, gastrointestinal, and nasal mucosal barriers where borneol could change the close connection between the epithelial cells, accelerate the transportation of drugs through the intercellular passage, increase the number and volume of pinocytosis vesicles in BBB cells, improve cell membrane permeability, change the integrity of the lipid cell membrane, decrease the collision and the resistance between phospholipid molecules, increase their fluidity, significantly inhibit the activity of drug resistance proteins such as MDR1 and P-gp, and then accelerated the transportation of drugs (Figure 6).

**Figure 7:**
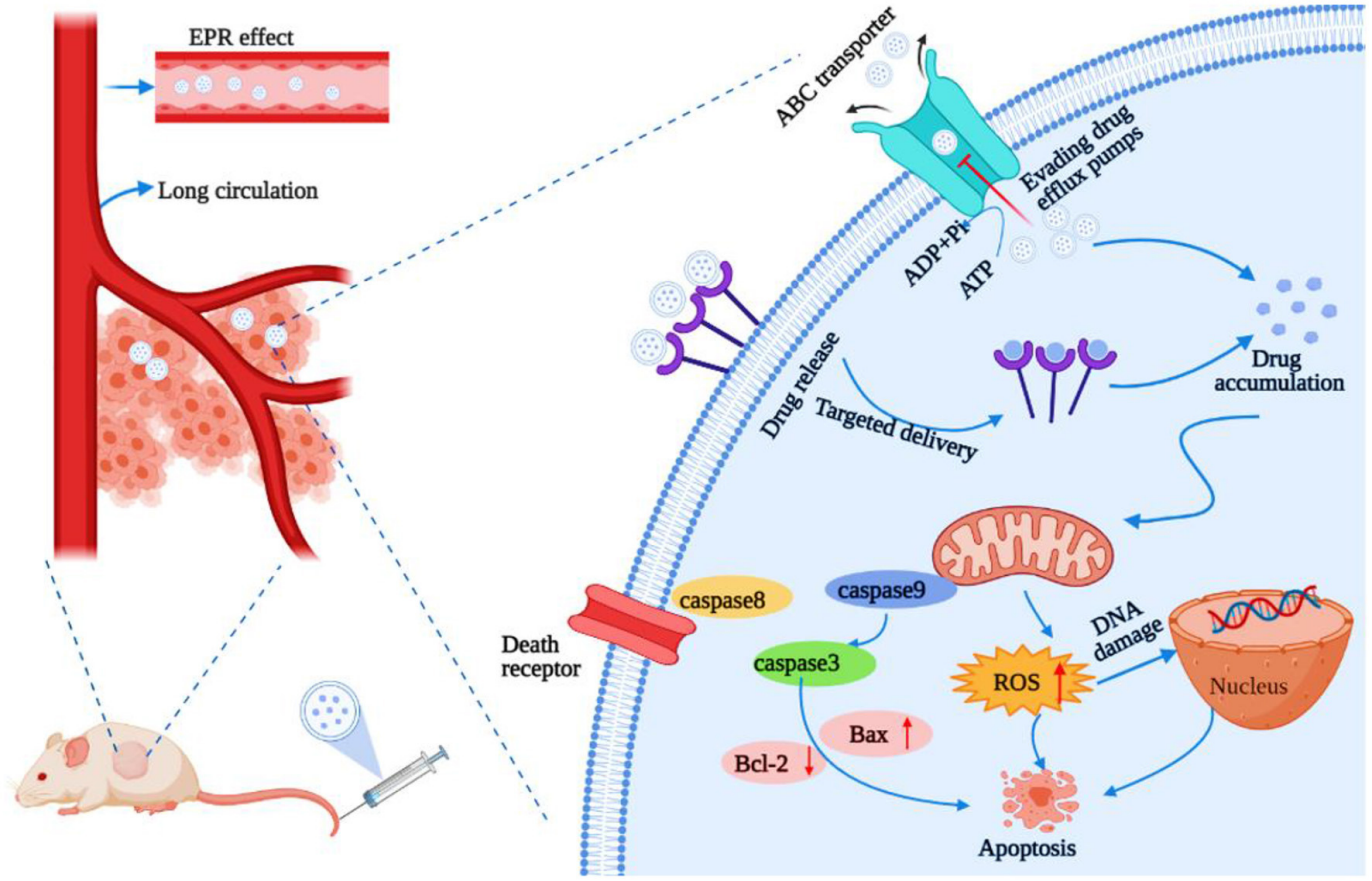
Main role and mechanism of borneol modified nanoparticle-based drug delivery platforms for overcoming multidrug resistance (MDR). The figure was created by BioRender, https://app.biorender.com/.

Borneol modified different types of nanocarriers, such as NPs, nanoemulsions, liposomes, dendrimers, polymer micelles, and lipoprotein nanocomposites applied in the drug delivery systems for treating solid tumors (lung, breast, brain, and liver cancer) could increase the solubility of drugs, enhance cellular uptake and cell cytotoxicity, decrease nanocarrier toxicity, reduce organ toxicity, and overcome MDR. Modified borneol could also enhance brain targeting, lymphatic targeting, drug distribution, and the antitumor effect. The main mechanisms include regulating the protein responsible for transporting drugs from the cell membrane and inhibiting their overexpression, including the ABC membrane transporter superfamily MRP1/ABCC1 and P-gp/ABCB1. Consequently, drug efflux is inhibited, drug entry into cells is promoted, and the accumulation and redistribution of therapeutic drugs at tumor target sites are increased, which, together, lead to the activation of signaling pathways, including those involved in ROS, DNA damage, and apoptosis (Figure 7).

## Future Perspectives

The above current studies strongly suggest that borneol modification nanocarriers have a promising future in the field of solid tumor treatment although the current research mostly stayed at the cell lines or the rodent-based models. The huge gap between the preclinical studies and human results remains to be filled. Furthermore, the safety of nanodrug accumulation in unwanted tissues and organs and the uncertain safety concerns of some nanomaterials need to be determined. The rational combination of multiple drugs with different modes of action is also a challenge, as are issues with manufacturing scale and practice, including reproducibility, non-uniform diameter, irregular structure/shape, sterilization, and storage for mass production (131). In addition, the relationship between the amount-time-effect and the effect of the immune system following borneol administration remains to be explored. Of course, with the in-depth understanding of tumor biology, the behavior of NPs in the body, the pharmacological mechanism of antitumor drugs, and the continuous discovery and clarification of the mechanism of herbs active ingredients, it is believed that the combination of traditional Chinese herbs and chemotherapeutic drugs in nanocarriers will bring surprises for tumor treatment and drug resistance. These improvements are more conducive to nanocarriers for releasing drugs at the correct place, time, and dose, and thereby improve efficiency and reduce toxic side effects. Only the cross-connection between different fields can make nanomedicine play a great role in improving efficacy, safety, and overcoming drug resistance, it will also be personalized according to the characteristics of the patient's disease to achieve precise treatment.

## Author Contributions

JL and JianW design and wrote the whole manuscript. QX, RM, YL, and JY carried out various literature survey studies. MR, HL, and JiajW participated in the drawing of the figures. DL and ZX checked the format initially. All authors contributed to the article and approved the submitted version.

## Funding

This work was supported by the National Natural Science Foundation of China (Grant Nos. 81873023 and 81473371), the Innovation Team in Chengdu University of Traditional Chinese Medicine (Grant No. CXTD2018004), and the Open Research Fund of the Key Laboratory of Southwestern Characteristic Chinese Medicine Resources, Chengdu University of Traditional Chinese Medicine (Grant No. 2020XSGG025).

## Conflict of Interest

The authors declare that the research was conducted in the absence of any commercial or financial relationships that could be construed as a potential conflict of interest.

## Publisher's Note

All claims expressed in this article are solely those of the authors and do not necessarily represent those of their affiliated organizations, or those of the publisher, the editors and the reviewers. Any product that may be evaluated in this article, or claim that may be made by its manufacturer, is not guaranteed or endorsed by the publisher.

## Abbreviations

PTX: paclitaxel
GIT: gastrointestinal tract
P-gp: P-glycoprotein
MDR: multidrug resistance
EPR: enhanced permeability and retention
NPs: nanoparticles
TCM: traditional Chinese medicine
BBB: blood-brain barrier
AUC: area under the curve
MRP1: multidrug resistance-related protein-1
NB: natural borneol
ROS: reactive oxygen species
NSCLC: Non-Small Cell Lung Carcinoma
BUN: blood urea nitrogen
AST: aspartate aminotransferase
TP: total protein
GLB: Globulin
A/G: albumin/globulin ratio
HDL-C: high-density lipoprotein cholesterol
ABC: ATP-binding cassette
AKT: protein kinase B
MAPK: mitogen-activated protein kinase
MRT: mean residence time
CL: clearance.
